# Genome-wide association study and genomic prediction in citrus: Potential of genomics-assisted breeding for fruit quality traits

**DOI:** 10.1038/s41598-017-05100-x

**Published:** 2017-07-05

**Authors:** Mai F. Minamikawa, Keisuke Nonaka, Eli Kaminuma, Hiromi Kajiya-Kanegae, Akio Onogi, Shingo Goto, Terutaka Yoshioka, Atsushi Imai, Hiroko Hamada, Takeshi Hayashi, Satomi Matsumoto, Yuichi Katayose, Atsushi Toyoda, Asao Fujiyama, Yasukazu Nakamura, Tokurou Shimizu, Hiroyoshi Iwata

**Affiliations:** 1 Laboratory of Biometry and Bioinformatics, Department of Agricultural and Environmental Biology, Graduate School of Agricultural and Life Sciences, The University of Tokyo, 1-1-1 Yayoi, Bunkyo, Tokyo 113-8657 Japan; 2 Institute of Fruit Tree and Tea Science, National Agriculture and Food Research Organization (NARO), 485-6 Okitsu Nakacho, Shimizu, Shizuoka 424-0292 Japan; 3 Genome Informatics Laboratory, National Institute of Genetics, Research Organization of Information and Systems, 1111 Yata, Mishima, Shizuoka 411-8540 Japan; 4 Institute of Fruit Tree and Tea Science, NARO, 2-1 Fujimoto, Tsukuba, Ibaraki 305-8605 Japan; 5 Institute of Crop Science, NARO, 2-1-2 Kannondai, Tsukuba, Ibaraki 305-8518 Japan; 6 Institute of Crop Science, NARO, 1-2 Ohwashi, Tsukuba, Ibaraki 305-8634 Japan; 7 Comparative Genomics Laboratory, National Institute of Genetics, Research Organization of Information and Systems, 1111 Yata, Mishima, Shizuoka 411-8540 Japan; 8 Advanced Genomics Center, National Institute of Genetics, Research Organization of Information and Systems, 1111 Yata, Mishima, Shizuoka 411-8540 Japan

## Abstract

Novel genomics-based approaches such as genome-wide association studies (GWAS) and genomic selection (GS) are expected to be useful in fruit tree breeding, which requires much time from the cross to the release of a cultivar because of the long generation time. In this study, a citrus parental population (111 varieties) and a breeding population (676 individuals from 35 full-sib families) were genotyped for 1,841 single nucleotide polymorphisms (SNPs) and phenotyped for 17 fruit quality traits. GWAS power and prediction accuracy were increased by combining the parental and breeding populations. A multi-kernel model considering both additive and dominance effects improved prediction accuracy for acidity and juiciness, implying that the effects of both types are important for these traits. Genomic best linear unbiased prediction (GBLUP) with linear ridge kernel regression (RR) was more robust and accurate than GBLUP with non-linear Gaussian kernel regression (GAUSS) in the tails of the phenotypic distribution. The results of this study suggest that both GWAS and GS are effective for genetic improvement of citrus fruit traits. Furthermore, the data collected from breeding populations are beneficial for increasing the detection power of GWAS and the prediction accuracy of GS.

## Introduction

Citrus are among the most produced fruits in the world^1^ and contain various functional compounds beneficial for human health such as vitamins, limonoids, and carotenoids^2^. Total citrus fruit production in 2012 was estimated at 131.3 million tons (20% of total fruit production; 8.8 kha), of which 52.6% were sweet oranges (*Citrus sinensis* (L.) Osbeck), 21.1% mandarins (*C*. *reticulata* Blanco), 11.2% lemons (*C*. *limon* (L.) Burm. f.) and limes (*C*. *aurantifolia* (Christm.) Swingle), and 6.2% grapefruit (*C*. *paradisi* Macfad.) and pummelos (*C*. *maxima* Merr.)^3^. Widely distributed commercial cultivars have been selected from various indigenous cultivars as somatic mutants (bud sports or nucellar seedlings)^4^. Although mutant selection is a simple approach to improve a trait, cross-breeding is beneficial for developing novel cultivars with unprecedented trait combinations. Swingle and Webber first initiated a systematic cross-breeding program of citrus at the U.S. Department of Agriculture in Florida, USA in 1893, and they selected some important cultivars by cross-breeding^5^. The available combinations of crosses and the number of individuals in the population limit the efficiency of selection. Large plant size limits the number of fruit trees in orchards, and their long juvenile period lengthens the time required for breeding, thus increasing the total cost of cross-breeding. During implementation of the citrus breeding program at the National Agriculture and Food Research Organization Institute of Fruit Tree and Tea Science (NIFTS) in Japan, various techniques to promote flowering and fruiting of the seedlings to shorten the breeding cycles and grafting and training methods were established^6, 7^; however, the average breeding period from the cross to the release of a cultivar is still 25 years. The number of seedlings to be grafted at the orchard is the key step limiting breeding efficiency. Therefore, selecting promising seedlings at an early stage before grafting would greatly increase the probability of obtaining promising candidates.

In recent years, next-generation sequencing technologies have decreased the cost of single nucleotide polymorphism (SNP) genotyping and expanded the availability of numerous markers^8^. Genome-wide association studies (GWAS) and genomic selection (GS), both performed by using genome-wide markers, are becoming important and effective tools for plant breeding^9, 10^. GWAS enable the detection of quantitative trait loci (QTLs) or causal genes from association between genome-wide markers and trait phenotypes, and outperform bi-parental QTL mapping because GWAS do not require the development of segregating populations^11, 12^. GS enables the selection of superior genotypes based on genomic estimated breeding values (GEBV) estimated from information on genome-wide markers, and is more effective than marker-assisted selection (MAS), especially for traits controlled by a large number of genes^13, 14^. In general, the power of GWAS and the accuracy of GS are boosted when sample size is large^11, 15^. However, accumulating large data sets sufficient for these analyses is difficult for fruit trees because of their long juvenile period, large plant size, and difficulties in phenotyping. Poland suggested the idea of “breeding-assisted genomics”, in which the data collected from actual breeding populations is used for functional genomics in plants^16^. In fruit tree breeding, phenotypic data accumulated every year in conventional breeding programs could be useful for GWAS and GS. Although a number of GWAS and GS studies have been reported in animals and plants^11, 13, 14^, the potential for using multiple actual breeding populations in combination with the parental population has not yet been explored.

The objective of this study was to assess the potential of GWAS and GS for the genetic improvement of citrus and to evaluate the potential of using multiple actual breeding populations in addition to the parental population for GWAS and GS.

## Results

### Linkage disequilibrium and population structure

**Figure 1:**
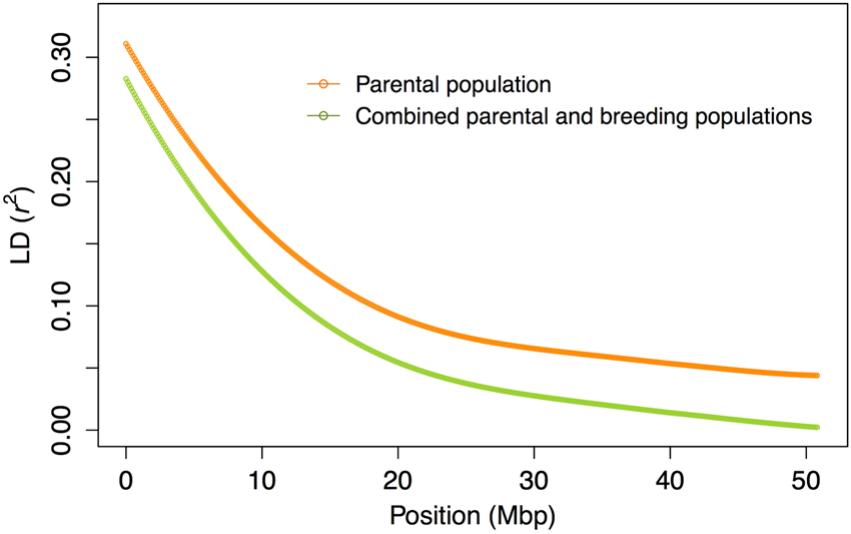
Linkage disequilibrium (LD) values (*r* ^2^) between pairs of SNPs plotted against physical distances between markers. Curves show local polynomial smoothed plots with kernel weight for the parental population (n = 111) and combined parental and breeding populations (n = 787).

The levels of linkage disequilibrium (LD) in the parental population (111 varieties; Supplementary Table S1) and combined parental and breeding populations (Supplementary Table S2; 787 genotypes in total) were evaluated by calculating the squared allele-frequency correlation (*r* ^2^) between each pair of SNPs on the same chromosome. The *r* ^2^ values were plotted against the physical distance for the parental population and the combined population; curves fitted for the relationships between *r* ^2^ and physical map distance indicated that LD extended over 1 Mb (*r* ^2^ > 0.25) and decayed to relatively low levels (*r* ^2^ < 0.10) within 20 Mb in both populations (Fig. 1). The mean LD between adjacent SNPs was similar (*r* ^2^ = 0.45) in both populations, although the decay of LD was slightly faster in the combined population. The patterns of LD decay were similar in all chromosomes (Supplementary Fig. S1).

**Figure 2:**
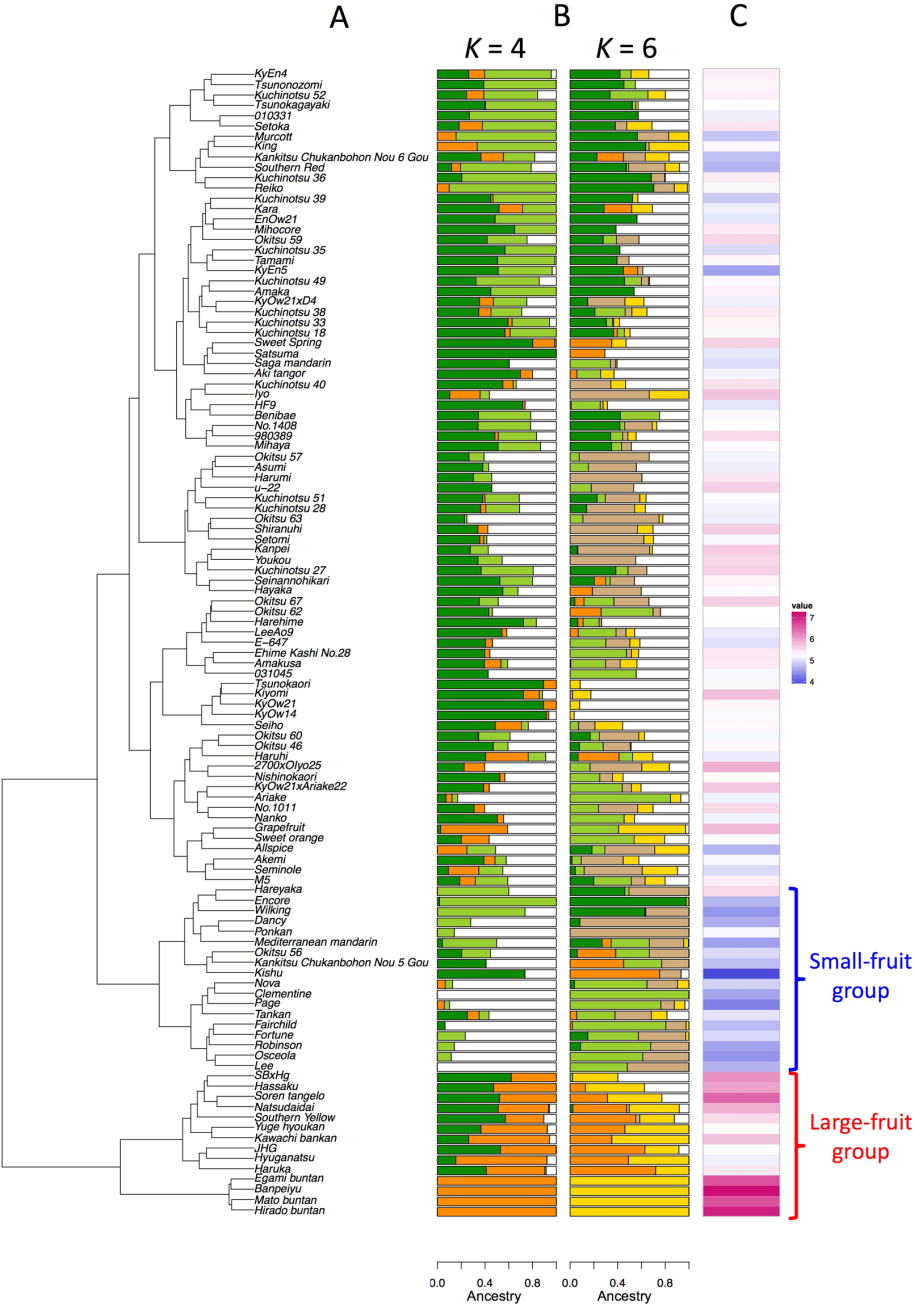
Genetic and phenotypic population structure of the parental population. (**A**) Ward’s hierarchical clustering based on Euclidean distance between genotypes. (**B**) ADMIXTURE-based estimation of the admixture proportions of individuals. Each color represents the inferred genetic contributions from *K* ancestral populations. (**C**) Heat map of log-converted fruit weight values.

The genetic structure of the parental population was estimated by using hierarchical clustering, ADMIXTURE clustering, and principal component analysis (PCA). Hierarchical clustering divided the parental population into two major clusters (Fig. 2A). The smaller cluster contained mainly pummelos, which are large-fruit varieties (Fig. 2C; Supplementary Table S1). The larger cluster was further divided into two major sub-clusters (Fig. 2A). The smaller sub-cluster contained mainly mandarins, which are small-fruit varieties (Fig. 2C; Supplementary Table S1). According to ADMIXTURE clustering, the optimal number (*K*) was estimated as 4, because cross-validation (CV) error rapidly decreased until *K* = 4 and changed little thereafter, although *K* = 6 corresponded to the lowest CV error (Supplementary Fig. S2). The deduced optimal *K* value was similar to those reported by Curk *et al*.^17^. The cluster containing large-fruit varieties but not the sub-cluster containing small-fruit varieties showed clear genetic differentiation (Fig. 2B). Similarly, PCA indicated the presence of two main clusters, one with large and the other one with small fruit size (Supplementary Fig. S3). Whereas part of the parental population belonged to the clusters of large- or small-fruit varieties, most varieties showed no clear separation in hierarchical clustering, ADMIXTURE clustering, or PCA. PCA also suggested low stratification in the combined population (Supplementary Fig. S4).

### Genome-wide association study

**Figure 3:**
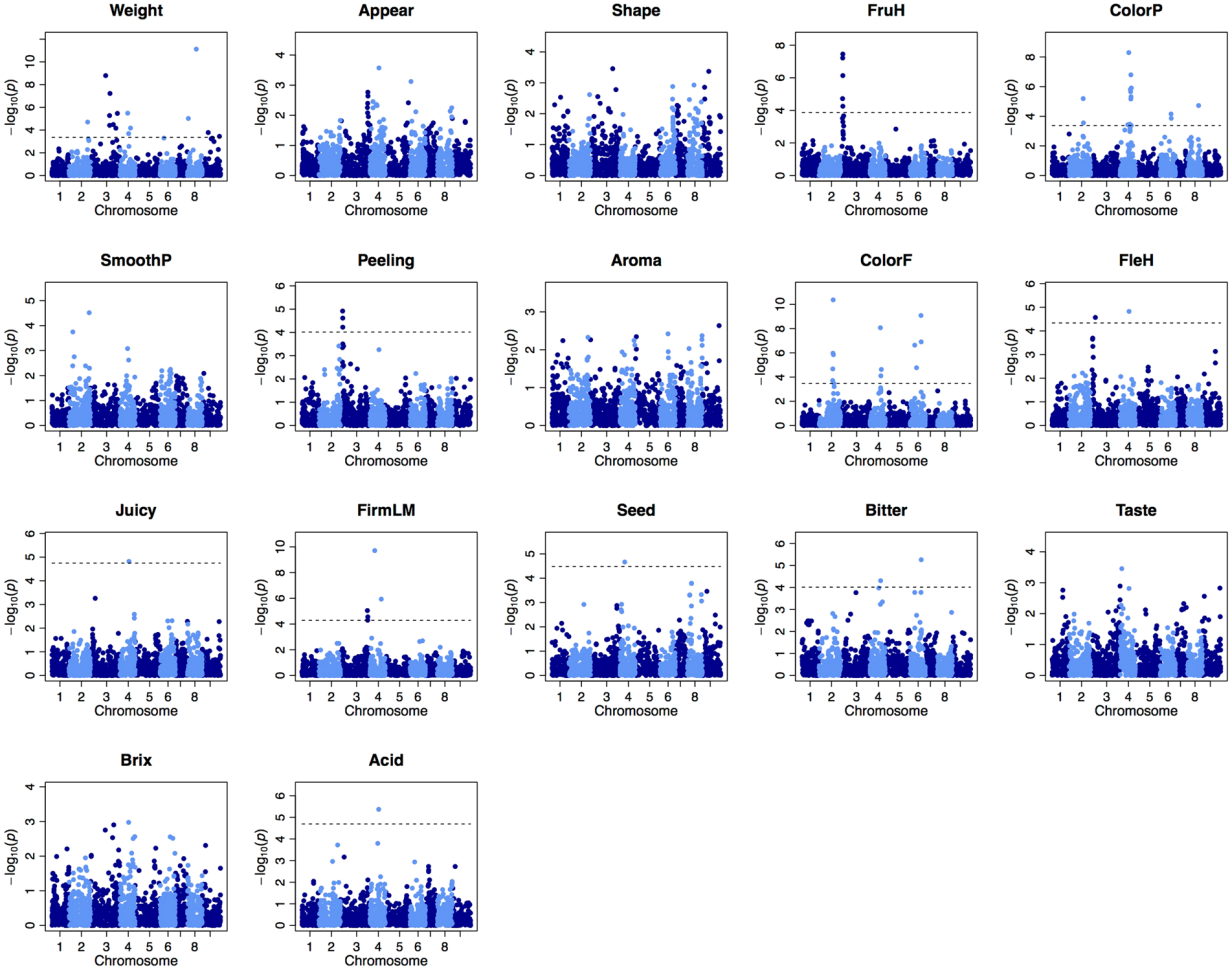
Manhattan plots for 17 fruit quality traits in the combined parental and breeding populations. Mixed linear model used four principal components of population structure as covariates (Supplementary Fig. S6). Dashed lines indicate a false discovery rate of 0.05.

GWAS was carried out for 17 fruit quality traits (Table 1) in the combined and parental populations (Fig. 3; Supplementary Table S3; Supplementary Figs S5 and S6). Higher –log_10_(*p*) values and/or more significant SNPs (false discovery rate <0.05) were detected for 15 traits (except for aroma intensity (Aroma) and bitterness (Bitter)) by using the combined population rather than the parental population. In the combined population, GWAS detected significant SNPs associated with 11 traits: fruit weight (Weight), fruit hardness (FruH), color of pericarp (ColorP), easiness of peeling (Peeling), color of flesh (ColorF), flesh hardness (FleH), juiciness (Juicy), firmness of locule membrane (FirmLM), number of seeds (Seed), Bitter, and acidity (Acid) (Supplementary Table S4). For the highly correlated phenotypic traits (*r* > 0.75; Supplementary Table S5), several common significant SNPs were detected: seven on chromosomes (Chr.) 2, 4, and 6 for ColorP and ColorF (*r* = 0.81) and three on Chr. 3 for FruH and Peeling (*r* = 0.76). Single common significant SNPs were detected for FleH and Juicy (Chr. 4; *r* = 0.68); FirmLM and Seed (Chr. 4; *r* = 0.35); and ColorP, ColorF, and Bitter (Chr. 6). A single common significant SNP for Weight, ColorP, Bitter, and Acid (Chr. 4) is consistent with the largest (although not significant) peak SNP for sugar content (Brix).

**Table 1:** Fruit quality traits evaluated in this study.

Trait	Abbreviation	Continuous or categorical value	Number of levels	Description
Fruit weight	Weight	Continuous	—	Fruit weight (g) (instrumental)
Appearance	Appear	Categorical	3	Good, intermediate, bad (sensory)
Fruit shape	Shape	Categorical	16	Very strongly oblate, strongly oblate, oblate, globose with truncated apex, globose, ellipsoid, ellipsoid with prominently nippled apex, pyriform, and each category with presence or absence of neck (including collar) at base (visual)
Fruit hardness	FruH	Categorical	5	Very soft, soft, intermediate, hard, very hard (sensory)
Color of pericarp	ColorP	Categorical	9	Green, cream, yellow, yellowish orange, light orange, orange, deep orange, light reddish orange, reddish orange (visual)
Smoothness of pericarp	SmoothP	Categorical	5	Smooth, moderately smooth, intermediate, moderately rough, rough (visual)
Easiness of peeling	Peeling	Categorical	5	Easy, moderately easy, intermediate, moderately difficult, difficult (sensory)
Aroma intensity	Aroma	Categorical	4	Strong, intermediate, weak, none (sensory)
Color of flesh	ColorF	Categorical	6	Cream, yellow, yellowish orange, light orange, orange, deep orange (visual)
Flesh hardness	FleH	Categorical	5	Very soft, soft, intermediate, moderately hard, hard (sensory)
Juiciness	Juicy	Categorical	3	Juicy, intermediate, dry (sensory)
Firmness of locule membrane	FirmLM	Categorical	5	Very soft, soft, intermediate, moderately hard, hard (sensory)
Number of seeds	Seed	Categorical	4	None (0), a few (1–2), intermediate (3–5), many (>6) (visual)
Bitterness	Bitter	Categorical	2	Present or absent (sensory)
Taste	Taste	Categorical	5	Very good, good, intermediate, bad, very bad (sensory)
Sugar content	Brix	Continuous	—	Total soluble solid content of juice (%) (instrumental)
Acidity	Acid	Continuous	—	Acidity of juice (%) (instrumental)

Two genes, Ciclev10031587 m.g and Ciclev10031003 m.g, were found between the significant SNPs on Chr. 4 for ColorP and ColorF; these genes were located 490 kb and 17 kb apart from the nearest significant SNPs, respectively, within a high-LD region (Supplementary Fig. S7). These genes are annotated as *phytoene desaturation 1* (*pds1*) and *carotenoid cleavage dioxygenase 4* (*ccd4*), respectively. A significant SNP on Chr. 3 for FruH and Peeling resided in the gene Ciclev10018456 m.g, which is annotated as *callose synthase*. This gene was also located in the region flanking a significant SNP for FleH. The common significant SNP on Chr. 4 for Weight, ColorP, Bitter, and Acid resided in the gene Ciclev10031681 m.g, which is annotated as *glutamate dehydrogenase*.

### Accuracy of prediction models for 17 fruit traits in the parental population

**Figure 4:**
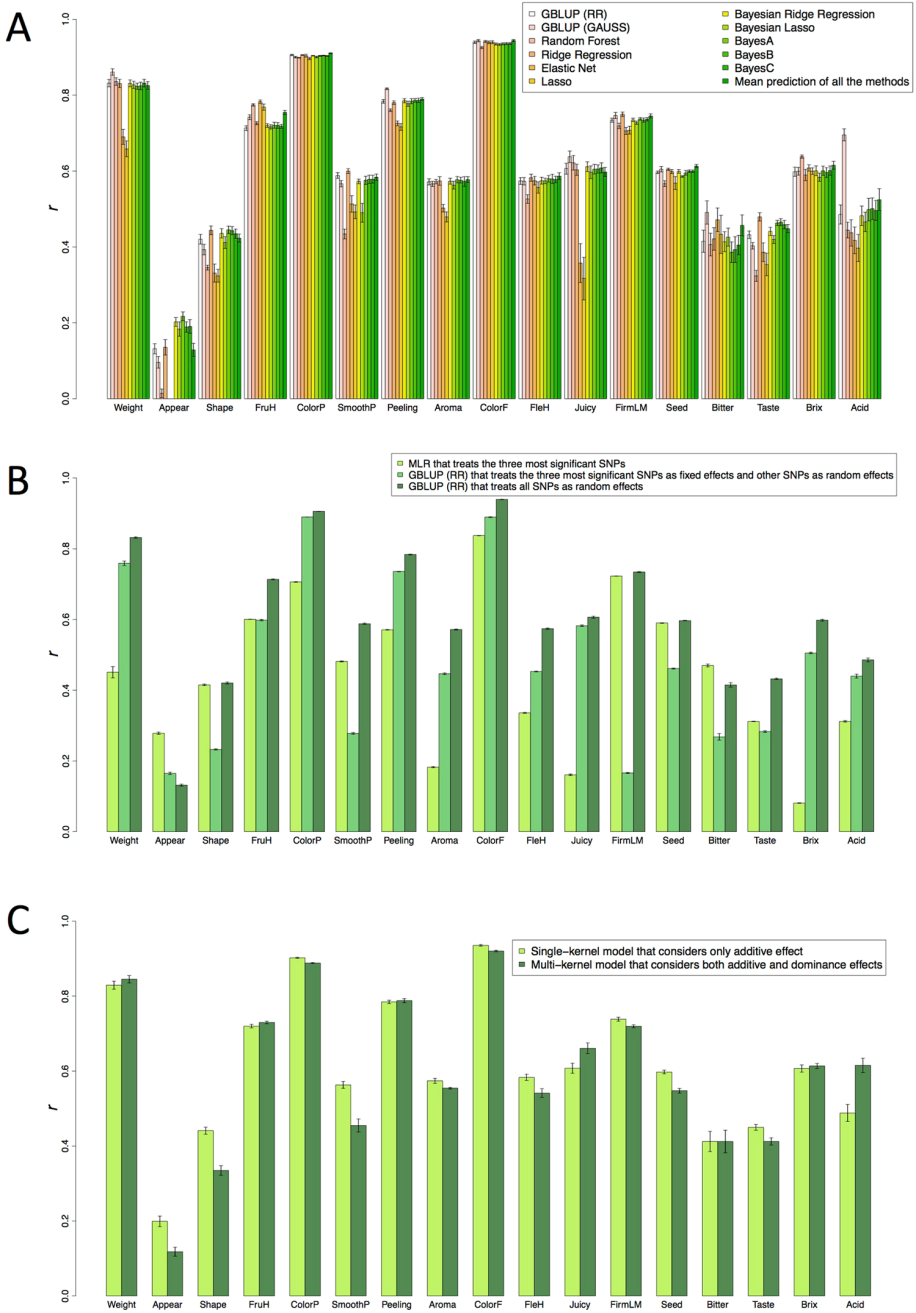
Comparison of prediction models using the parental population. Prediction accuracy was measured as the Pearson’s correlation coefficient (*r*) between predicted genotypic values and phenotypic values. (**A**) Twelve methods were tested. RR: ridge kernel regression, GAUSS: Gaussian kernel regression. (**B**) Regression models were built based on the results of GWAS. The three SNPs that showed high –log_10_(*p*) values in GWAS (red points in Supplementary Fig. S5) were selected. MLR: Multiple Leaner Regression. (**C**) Prediction models that considered only additive or both additive and dominance effects were used.

Ten-fold CV showed that prediction accuracy was high for Weight, FruH, ColorP, Peeling, ColorF, and FirmLM (*r* ≥ 0.7), intermediate for fruit shape (Shape), smoothness of pericarp (SmoothP), Aroma, FleH, Juicy, Seed, Bitter, taste (Taste), Brix, and Acid (0.3 ≤ *r* < 0.7), and low for appearance (Appear) (*r* < 0.3) (Fig. 4A). GBLUP (RR and GAUSS), Ridge Regression, and Bayesian regressions were more accurate for most traits than the other methods, whereas the accuracies of Random Forest, Elastic Net, and Lasso were trait-dependent. While the prediction accuracy level of a single method considerably varied among traits, the mean prediction of all 11 methods showed consistent accuracy, i.e., an upper-middle level of accuracy among the methods, for all traits. The non-linear method GBLUP (GAUSS) had the highest prediction accuracy for Acid. Because most of the traits (except Weight, Brix, and Acid) were scored as ordinal categorical traits, several prediction models specific for categorical responses were used. Because the prediction accuracy of these models was lower than that of models for continuous responses (Supplementary Fig. S8), the latter models were used for subsequent analyses.

We built prediction models that included the information from GWAS for the 17 traits. For 15 traits (except Appear and Bitter), GBLUP (RR) that treats all SNPs as random effects was more accurate than multiple linear regression (MLR) that treats the three most significant SNPs (*r* < 0.6; red points in Supplementary Fig. S5) as fixed effects, or GBLUP (RR) that treats the three most significant SNPs as fixed effects and other SNPs as random effects, even though MLR had high prediction accuracies (*r* > 0.7) for ColorP, ColorF, and FirmLM (Fig. 4B).

A multi-kernel model considering dominance and additive genetic effects was also evaluated. For Acid and Juicy, this model was more accurate than a model with only additive effects, whereas opposite results were obtained for Appear, Shape, SmoothP, FleH, Seed, and Taste (Fig. 4C). For the other traits, accuracies were similar for both models. The additive genetic effects were the major factor contributing to genetic variation for 14 traits (except Appear, Juicy, and Bitter) (Supplementary Table S6).

### Prediction for distribution tails

**Figure 5:**
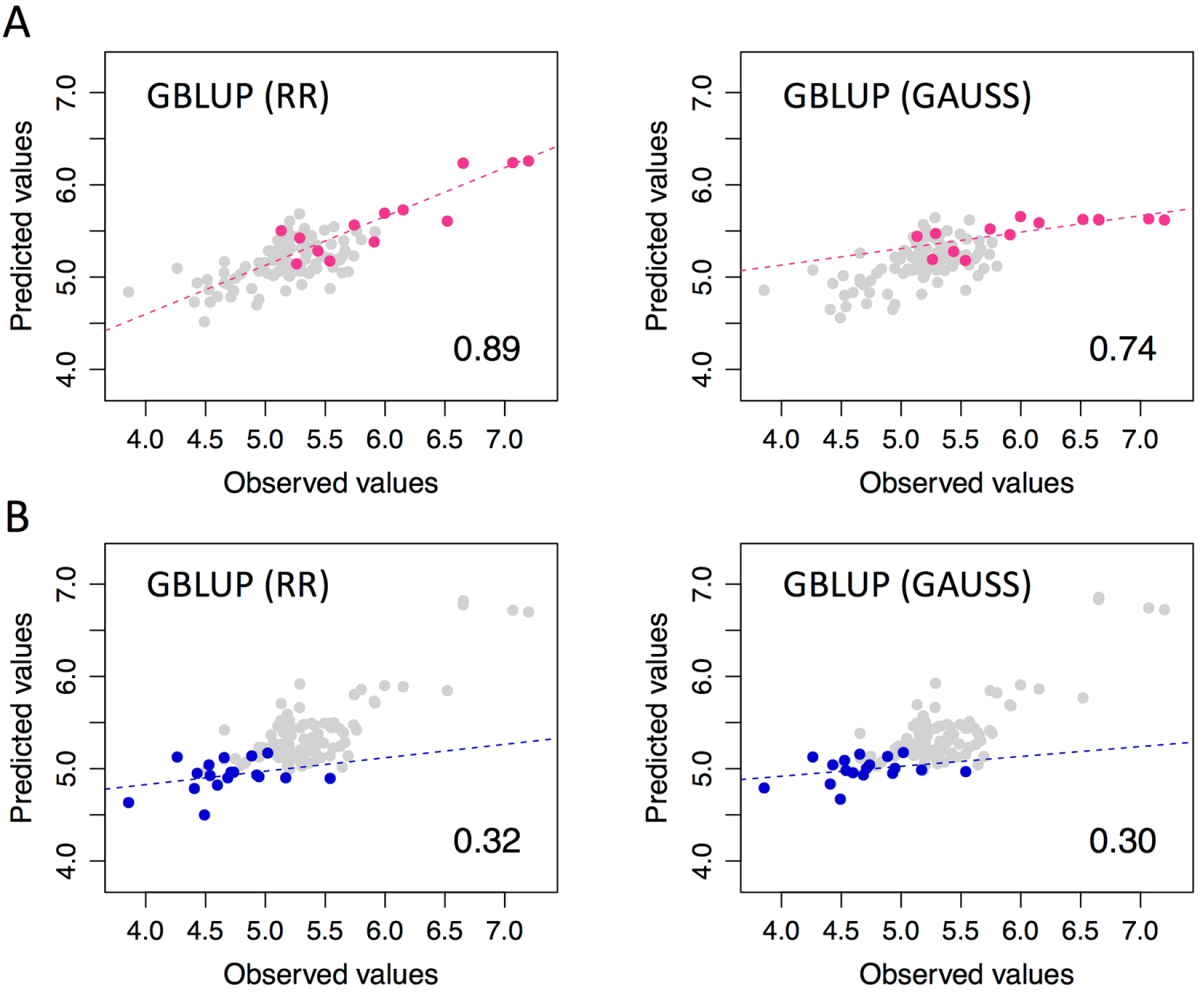
Prediction for fruit weight distribution tails. (**A**) All varieties except those in the large-fruit group (Fig. 2C), which is indicated by pink points, were used as the training set of the prediction model; the large-fruit group was used as the test set. (**B**) All varieties except those in the small-fruit group (Fig. 2C), which is indicated by blue points, were used as the training set; the small-fruit group was used as the test set. Grey points indicate the results of leave-one-out cross-validation. Predicted and observed values indicate log-transformed Weight. Pearson’s correlation coefficient (*r*) between predicted and observed values for the pink or blue points are shown on the plots. RR: linear ridge kernel regression, GAUSS: non-linear Gaussian kernel regression.

As described above, a diversity of fruit weight was found in the parental population (Fig. 2; Supplementary Fig. S9A). Because the fruit weight of pummelos, such as ‘Egami buntan’, ‘Banpeiyu’, ‘Mato buntan’, and ‘Hirado buntan’, was very large (>800 g), we hypothesized that a genetic system differs between the varieties with extremely large fruits and other varieties. To validate the hypothesis, we tried to predict fruit weight in the large-fruit group (Fig. 2) by using a model built based on the data of other citrus varieties. The results showed a high accuracy of such prediction (Fig. 5A). Higher accuracy was attained by GBLUP (RR) (*r* = 0.89) than by GBLUP (GAUSS) (*r* = 0.74), whereas the accuracy of GBLUP (RR) was slightly lower than that of GBLUP (GAUSS) for all ranges of fruit weight (Fig. 4A). The extremely large fruit weight in pummelos was properly predicted, especially with GBLUP (RR). Similarly, GBLUP (RR) had a slightly higher accuracy for the small-fruit group (Fig. 5B). We also applied rank-ordered 4-fold CV for all 17 traits. GBLUP (RR) attained higher accuracy than did GBLUP (GAUSS) (including the number of outliers) for distribution tails for all the traits (Supplementary Fig. S9).

### Prediction for the breeding population

**Figure 6:**
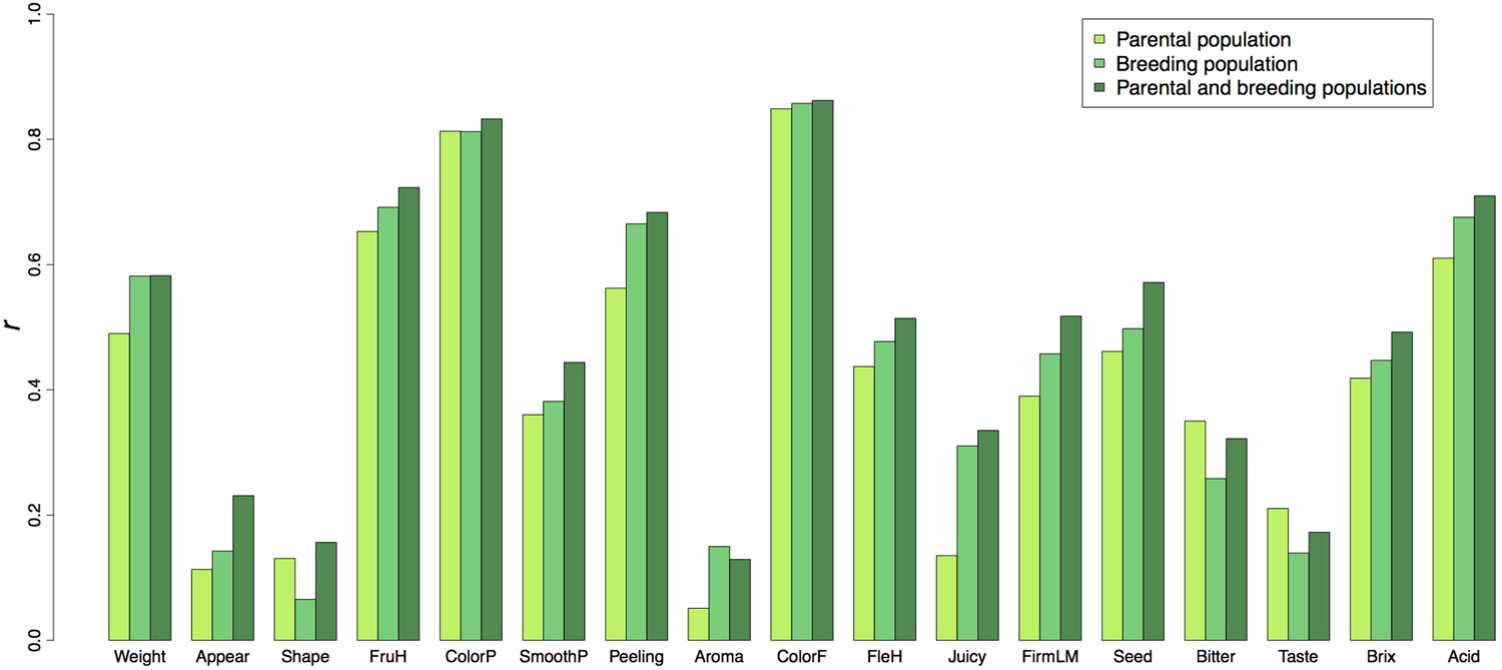
Prediction accuracy of the breeding population. Prediction accuracy was measured as the Pearson’s correlation coefficient (*r*) between predicted genotypic values and phenotypic values. The prediction accuracy was calculated for combined all families. Only the mean prediction accuracy of all the methods is shown (Supplementary Fig. S10). Training populations are shown in the figure key. When the breeding population or combined parental and breeding populations were used as training sets, one family was excluded and its phenotype was predicted.

To evaluate the prediction accuracy in the breeding population, three different training populations for modeling were compared. For 13 traits (except Shape, ColorP, Bitter, and Taste; Fig. 6, Supplementary Fig. S10), the accuracy of the model based on the breeding population was on average 1.35 times that of the model based on the parental population, indicating that information from the breeding population improved prediction accuracy. The highest accuracy was attained in the model based on the combined population for 14 traits (except Aroma, Bitter, and Taste). The results were similar when prediction accuracy was evaluated on a single-family basis (Supplementary Fig. S11).

## Discussion

The LD pattern is a key factor influencing the power of GWAS and the accuracy of GS, because these two approaches are based on LD between markers and causal polymorphisms^9, 11, 13, 18^. Generally, the range of LD is smaller in heterozygous outcrossing species than in homozygous selfing species^19, 20^, and therefore a large number of markers are needed to cover the genome of outcrossing species. In the citrus population used in this study, we observed a wide range of LD (*r* ^2^ > 0.25 at 1 Mb; Fig. 1), even though most genotypes obtained from conventional cross-breeding are highly heterozygous^21^. Because the citrus population has experienced a population bottleneck, i.e. it had a limited number of founders^3^, the wide range of LD^19, 20^ was expected to increase. Citrus cultivars are developed through a small number of generations, and are mainly maintained through vegetative propagation. These breeding and management systems make recombination unlikely to occur and may also maintain LD^22^. The mean *r* ^2^ between adjacent markers has been used to determine target marker densities for GS. For high-heritability traits, a mean *r* ^2^ of 0.15 is sufficient, but for low-heritability traits increasing the *r* ^2^ to 0.2 improved prediction accuracy^23^. In the citrus population used in this study, mean *r* ^2^ (0.45) was higher than the above value, suggesting that marker densities in the population would be sufficient for GS.

Population structure extends the range of LD^24^ and can cause spurious associations in GWAS^11^. Hierarchical clustering, ADMIXTURE clustering, and PCA showed that some varieties in the parental population formed clusters related to fruit weight, but most varieties showed no clear separation. The wide range of LD observed in the parental population was probably due to its small size rather than to the population structure. Given the wide range and high level of LD and the weak population structure, GWAS and GS would be effective in citrus populations.

For most traits, the number of significant SNPs and the –log_10_(*p*) values of peaks detected in GWAS were greater with the combined (787 genotypes in total) than with the parental population (111 varieties), which would increase the chance of finding good markers for MAS. These results indicate that GWAS using the combined population has higher statistical power for detecting QTLs than GWAS using only the parental population, and that the QTLs detected were shared between the parental and breeding populations. For some traits, the significant SNPs differ between the combined and parental populations, possibly because of the differences between the populations in detection power, allele frequency, or population structure.

In addition to the sample size, the difference in the LD pattern between the combined and parental populations can influence the resolution of GWAS. For example, multi-breed GWAS had a higher resolution than within-breed GWAS in dairy cattle^25^. This difference was explained by narrower QTL regions because of the short conserved regions of LD across breeds^26^. In the present study, the LD decay was rapid in the combined population, although the mean LD between adjacent SNPs was similar (*r* ^2^ = 0.45) in both populations, implying that the LD in the combined population is conserved over shorter distances than that in the parental population. The short conserved regions of LD could improve the precision of QTL detection in the combined population by narrowing down the QTL regions.

GWAS in the combined population detected SNPs significantly associated (false discovery rate <0.05) with 11 traits. The most significant association was detected for Weight on Chr. 8 where one QTL named *FW8* for fruit weight has been identified in a bi-parental QTL mapping study that used a mandarin F_1_ population derived from ‘Fortune’ × ‘Murcott’^27^. The most significant SNP detected for Weight resided in the same region as *FW8*, confirming a single major QTL for fruit weight on Chr. 8.

Identification of seven common significant SNPs for ColorP and ColorF suggests a close genetic linkage between QTLs controlling these traits, resulting in high phenotypic correlation. Common significant SNPs for other traits could be explained similarly. Significant SNPs for ColorP and ColorF on Chr. 4 were located in multiple QTLs for fruit flavedo and juice color identified on the linkage group MUR4.1^27^. Two genes, Ciclev10031587 m.g and Ciclev10031003 m.g, annotated as *pds1* and *ccd4* respectively, are colocalized with the QTLs on MUR4.1^27^. The *pds1* gene is involved in the carotenoid biosynthetic pathway in citrus^28, 29^ and Arabidopsis^30^, whereas *ccd4* is involved in carotenoid accumulation in peach^31, 32^. The pericarp and flesh color of citrus fruits is determined by carotenoid accumulation^3^.

A significant SNP for FruH and Peeling on Chr. 3 resides in the *callose synthase* gene (Ciclev10018456 m.g). The polysaccharide callose is a component of plant cell wall^33^. Cell wall polysaccharides are associated with cell wall elasticity and viscosity, which influence fruit firmness. Softening of fruit tissues in tomato and avocado is caused by the degradation of cell wall polysaccharides^34, 35^.

A single significant SNP for Acid detected on Chr. 4 is consistent with those for Weight, ColorP, and Bitter, and with the largest (although not significant) peak SNP for Brix. This SNP resides in the gene Ciclev10031681 m.g, which is annotated as *glutamate dehydrogenase*. In avocado, the activity and protein content of GDH remain constant during fruit development, but increase ~4-fold during fruit ripening^36^. Generally, the acidity decreases while the sugar content increases during fruit maturation^3^. GDH could have pleiotropic effects on fruit quality traits involved in citrus fruit maturation. If so, common significant SNPs could be used as common markers for such traits in MAS programs.

The prediction accuracy of GBLUP (RR) that treats all SNPs as random effects, was highest for all the traits except Appear and Bitter, implying that all the traits except Appear and Bitter are controlled by many genes and/or QTLs. For Shape, SmoothP, Aroma, Taste, and Brix, for which the levels of prediction accuracy were intermediate, even though no significant association was detected. These results suggest that GS is more accurate for these traits than MAS based on significant SNPs. On the other hand, high prediction accuracies (*r* > 0.7) of MLR for ColorP, ColorF, and FirmLM imply that MAS performed by using a few markers enables the selection at low-cost genotyping for these traits.

A multi-kernel model that considers both additive and dominance effects improved the accuracy for Acid and Juicy, suggesting that the effects of both types are important for these traits. The highest prediction accuracy of GBLUP (GAUSS) for Acid supports this idea. Whereas GBLUP (RR) captures additive genetic effects, GBLUP (GAUSS) captures non-additive genetic effects (e.g., the dominance effect) by modeling non-linear relationships between markers and the phenotype^37, 38^. For the other traits, prediction accuracy was similar or lower with the model considering additive and dominance effects, suggesting that additive models are sufficient to explain genetic variations in the population. Consistent with this assumption, additive genetic effects were the major factor contributing to genetic variation. Using models that consider both additive and dominance effects may not always be beneficial, as suggested by several previous studies. Adding dominance and epistatic effects to additive genetic effects did not help genomic prediction in rice, suggesting a high correlation between multiple variance components^39^. In wheat, considering only additive effects resulted in equal or even higher prediction accuracy than considering both additive and dominance effects^40^, even though considering both types of effects increased the prediction accuracy in another simulation study that used a large population^41^. These results suggest that models considering dominance effects are quite sensitive to the number of individuals in the population^40^.

The prediction for the tails of fruit weight distribution was accurate for large-fruit varieties. The extremely large fruit weight of pummelos was properly predicted, especially with GBLUP (RR), even though the accuracy of GBLUP (RR) for the whole range of distribution was slightly lower than that of GBLUP (GAUSS). This implies that the genetic architecture of the extremely large fruit weight of pummelos can be explained by the additive accumulation of fruit weight–related genes of “non-large-fruit” varieties, which include a number of mandarins and sweet oranges. Cultivated pummelos were originally selected from a single progenitor species, *C*. *maxima*, whilst some of the cultivated mandarins were developed by introgression from *C*. *maxima* to the ancestral mandarin species *C*. *reticulata* ^42^; sweet oranges (*C*. *sinensis*) originated from pummelo and mandarin^43^. Thus, mandarins and sweet oranges are related to pummelos, which may have contributed to the high prediction accuracy for the extremely large fruit weight of pummelos. For other traits, RR also outperformed GAUSS in the prediction of the distribution tails, suggesting that RR is much more robust and accurate than GAUSS when used for this purpose. This is an important implication for the use of GS in breeding programs, because the prediction of distribution tails is important for increasing or decreasing genotypic values of target traits.

For the breeding population, the prediction accuracy was lower in the model built using only the parental population than in the model built using the combined population. This result suggests that the high genetic diversity in the parental population is not always useful to predict a small variation in the breeding population. Generally, the prediction accuracy across populations that are of low relevance is lower than that within a population^44^. Lower prediction accuracies across populations than within populations were also observed in apple^45, 46^, grapevine^47^ and maize^48^. On the other hand, across-population evaluation is reportedly preferable to that within populations when the populations are closely related to each other, marker density is high, or the number of phenotypic records is small^49, 50^. The advantage of using data from multiple populations should be discussed from the viewpoint of the costs of genotyping and phenotyping, as pointed out by Muranty *et al*.^46^. In the present study, the prediction accuracy was improved by combining the parental and breeding populations. The shorter conserved region of LD in the combined population than in the parental population could improve not only GWAS resolution but also the prediction accuracy by narrowing down the QTL regions. Our results suggest that multiple populations closely related to each other are useful to predict a small variation in the breeding population.

The results of this study suggest that GWAS and GS have a good potential for genetic improvement of fruit quality traits in citrus. Declining sequencing cost enables genomics-assisted breeding with very large numbers of DNA markers, and it has been anticipated that this approach would improve fruit tree breeding by avoiding constraints imposed by the long juvenile period and large plant size^51^. In the present study, the GWAS power was increased and the prediction accuracy was improved by combining the breeding and parental populations. The results support the idea of breeding-assisted genomics, in which the data collected from actual breeding programs will be beneficial for plant functional genomics^16^. Because the phenotypic data are accumulated every year, combining marker genotypic data in a breeding program and gathering them will increase the detection power of GWAS and the prediction accuracy of GS. The combination of genomics-based approaches, GWAS and GS, and the data from actual breeding programs will facilitate both functional genomics and fruit tree breeding.

## Methods

### Plant materials and fruit assessment

A set of 111 citrus varieties (48 hybrid cultivars, 41 breeding/selected strains, 22 indigenous varieties), called the parental population (Supplementary Table S1), and 35 full-sib families with a total of 676 F_1_ individuals, called the breeding population (Supplementary Table S2), were used in this study. The breeding population was derived from crosses among 45 varieties, of which 41 were included in the parental population. All genetic materials were maintained at NIFTS (Nagasaki and Shizuoka, Japan).

We evaluated 17 fruit quality traits for the parental and breeding populations by using instrumental, sensory, or visual assessment methods (Table 1). Five fruits were sampled from one tree of each genotype in December of each year, when many genotypes were maturing. The mean values obtained from 2008 to 2014 were estimated by fitting a mixed linear model (MLM); year was treated as a fixed effect and variety as a random effect to remove the year effect. The best linear unbiased prediction (BLUP) estimated by the MLM was used for subsequent analysis as the dependent variables of regression models for GWAS and genomic prediction models. The MLM was implemented in the “lmer” function of the R package lme4 ver. 1.1–7^52^. All traits except Weight, Brix, and Acid had ordinal categorical values (Table 1).

### SNP genotyping data

DNA was extracted and its quality was evaluated according to Shimizu *et al*.^53^. Next-generation sequencing data of 12 citrus varieties was obtained with Illumina HiSeq 2000 in paired-end mode for the development of a SNP Array for genotyping 768 SNPs^53^, and a SNP array for genotyping 1,536 SNPs was designed from the sequencing data as described by Shimizu *et al*.^53^.

SNP genotyping for 768 or 1,536 arrays was performed as described by Shimizu *et al*.^53^. Parentage tests were performed using a function of GUGS (General Utilities for Genotyping Study) software (T. Shimizu, *in preparation*) with known hybrid trios. Finally, 1,841 verified SNPs were obtained (Supplementary Data S1). For further analysis, each of the 1,841 SNP genotypes was converted to 1 (AA homozygotes), −1 (BB homozygotes), or 0 (AB heterozygotes). The rate of missing SNP genotypes was 0.17. We used BEAGLE ver. 3.3.2^54^ to impute the missing genotypes; the mean imputation accuracy (*r* ^2^) was 0.97.

### Estimation of linkage disequilibrium

To estimate LD between each pair of SNPs within the same chromosome, we calculated the squared correlation coefficients (*r* ^2^) of SNP genotypes and plotted them against physical distance between the corresponding markers in Mb. Local polynomial regression with kernel weight was conducted using the “locpoly” function in the R package KernSmooth ver. 2.23–13^55^ to test the relation between the *r* ^2^ values and physical map distances. Physical distances between adjacent markers were 0.056–2,243 kb (mean, 156.4 kb).

### Population structure analysis

The genetic structure of the parental population was estimated using hierarchical clustering, ADMIXTURE clustering, and PCA. Hierarchical clustering based on Ward’s method^56^ with Euclidean distance was conducted by using the R function “hclust”. Model-based clustering implemented in the software ADMIXTURE ver. 1.3.0^57^ infers population structure by estimating individual admixture proportions from multi-locus SNP data by using a maximum-likelihood method. The number of ancestral populations (*K*) in the parental population varying from 1 to 10 was assumed when using ADMIXTURE. To choose the optimal *K* value, 5-fold CV was performed. PCA was conducted using the R function “prcomp”. The optimal number of principal components (PCs) in the parental population was determined by estimating the variances of PC scores. To locate the breeding population in the PCA space of the parental population, the PC scores of the breeding population were estimated based on PCA of the parental population. A heat map was generated to estimate the phenotypic population structure of fruit weight (log-converted Weight) by using R package ggplot2 ver. 1.0.0^58^.

### Regression models for GWAS

Association analysis was conducted by using an MLM^59^ implemented in the “GWAS” function of the R package rrBLUP ver. 4.3^37^. To avoid spurious associations due to population structure, a kinship matrix and four PCs were included in the MLM as fixed effects. The kinship matrix was computed by using the “A.mat” function of the R package rrBLUP ver. 4.3^37^. The number of PCs was selected based on the variances of PC scores. The variance of PC score rapidly decreased until PC4, and gradually thereafter (Supplementary Fig. S6). Annotations of the genes containing significant SNPs were obtained from the Phytozome version 12.0.1 (https://phytozome.jgi.doe.gov/pz/portal.html/) and CitrusCyc Pathways version 3.0 databases (http://pathways.citrusgenomedb.org/). The LD heatmaps of the regions surrounding the peaks on Chr. 4 for ColorP and ColorF were constructed by using the R package LDheatmap ver. 0.99–1^60^.

### Genomic prediction models

We used 12 methods to evaluate prediction accuracy. A MLM-based method, genomic best linear unbiased prediction (GBLUP), was performed by using the “kinship.BLUP” function of the R package rrBLUP ver. 4.3^37^ with linear ridge kernel regression (RR) and non-linear Gaussian kernel regression (GAUSS). We also used the R package glmnet ver. 1.9–8^61^ for three different linear regression methods: Ridge Regression (alpha = 0), Lasso (alpha = 1), and Elastic Net (alpha = 0.5). The latter is a combination of ridge regression and lasso. A non-linear decision tree-based ensemble learning method, Random Forest, was run in the R package randomForest ver. 4.6–10^62^. The Bayesian linear regression models Bayesian Ridge Regression (BRR), Bayesian Lasso (BL), BayesA, BayesB, and BayesC were also used for the modeling. These models were implemented in the R package BGLR ver. 1.0.3^63^.

Because 14 of the 17 traits analyzed in this study were scored as ordinal categories (Table 1), seven regression models for the categorical data were also tested. The categorical data was obtained by rounding off the mean continuous values. We used the ordinal probit model-based BayesA and BayesB^64, 65^, and the R package BGLR for BRR, BL, BayesA, BayesB, and BayesC. The BGLR package supports models for categorical traits.

To evaluate the effect of peak SNPs with high –log_10_(*p*) values detected in GWAS, the accuracies of three models were compared. The first model was MLR implemented in the R function “lm”^66, 67^. The top three peak SNPs with high –log_10_(*p*) values of each trait were entered in MLR unless the correlation coefficient between the SNPs was ≥0.6 to prevent multicollinearity. The proportions of the variance explained by the top three peak SNPs for each trait were also estimated by the MLR model. The second model was GBLUP (RR) that treats the effects of the top three SNPs as fixed effects and the effects of other SNPs as random effects. The last model was the commonly used version of GBLUP (RR), which treats all SNPs as random effects.

To take into account the high observed heterozygosity (*H*_o_) of the parental population (>0.50)^21^, a multi-kernel model that considers both additive and dominance genetic effects was compared with a single-kernel model that considers only additive effects. Both models were implemented in the R package of BGLR ver. 1.0.3^63^. The additive relationship matrix (kinship matrix) was computed by the “A.mat” function of the R package rrBLUP, whereas the dominance relationship matrix was calculated as *D* in equation (6) in Vitezica *et al*.^68^ using a modified “A.mat” function. The additive (${\sigma }_{a}^{2}$) and dominance (${\sigma }_{d}^{2}$) genetic variances and residual variance (${\sigma }_{e}^{2}$) were estimated by using the multi-kernel model. Narrow-sense heritability (*h* ^2^) of each trait was computed as the ratio of ${\sigma }_{a}^{2}$ to the total phenotypic variance $({\sigma }_{a}^{2}+{\sigma }_{d}^{2}+{\sigma }_{e}^{2})({h}^{2}={\sigma }_{a}^{2}/({\sigma }_{a}^{2}+{\sigma }_{d}^{2}+{\sigma }_{e}^{2}))$.

To verify the models, we evaluated the prediction accuracy by 10-fold CV using the parental population randomly partitioned into each fold. The CV was repeated 5 times, and the same partition patterns were adapted to all prediction models in each CV. The prediction accuracy was defined as Pearson’s correlation coefficients (*r*) between observed and predicted genotypic values and regarded as *r* = 0 at *r* < 0.

To evaluate the prediction accuracy for the tails of the distribution of fruit weight, we employed the linear ridge kernel regression model GBLUP (RR) and the non-linear Gaussian kernel regression model GBLUP (GAUSS). All varieties except the large- and small-weight groups were used as a training set, whereas the large- and small-weight groups were used as a test set to assess the difficulty of the prediction of the distribution tails. The training set was also used for leave-one-out CV as a control. The prediction accuracy for the distribution tails was also evaluated for other traits. Rank-ordered 4-fold CV of the parental population was performed. The prediction accuracy was calculated in each partition. To show the tails of the distribution, phenotypic variations of the traits were visualized as jitter plots superimposed onto boxplots by using the R packages ggplot2 ver. 1.0.1^58^ and Rmisc ver. 1.5^69^.

To evaluate the prediction accuracy in the breeding population, all 12 methods were applied. Three different training populations for modeling were compared: only the parental population, only the breeding population (with one family excluded), and both the parental and breeding populations combined (with one family excluded); the phenotype of the excluded family was predicted to mimic actual breeding in which the phenotypic information of a family targeted by GS is generally not available. The prediction accuracies for each family or combined all families were calculated.

## Electronic supplementary material

Supplementary information

Dataset 1
