# Supplementary material for: Genome-wide association study and genomic prediction in citrus: Potential of genomics-assisted breeding for fruit quality traits
Source: Sci Rep. 2017 Jul 5;7:4721. doi: 10.1038/s41598-017-05100-x (PMC5498537; doi:10.1038/s41598-017-05100-x)
Supplement: Supplementary file 1 — Supplementary information [file 41598_2017_5100_MOESM1_ESM.pdf]

Supplementary information

Genome-wide association study and genomic prediction in citrus: Potential of genomics-assisted breeding for fruit quality traits

Mai F. Minamikawa[1], Keisuke Nonaka[2], Eli Kaminuma[3], Hiromi Kajiya-Kanegae[1], Akio Onogi[1], Shingo Goto[2], Terutaka Yoshioka[2], Atsushi Imai[4], Hiroko Hamada[2], Takeshi Hayashi[5], Satomi Matsumoto[6], Yuichi Katayose[6], Atsushi Toyoda[7,8], Asao Fujiyama[8], Yasukazu Nakamura[3], Tokurou Shimizu[2], Hiroyoshi Iwata[1*]

[1]Laboratory of Biometry and Bioinformatics, Department of Agricultural and Environmental Biology, Graduate School of Agricultural and Life Sciences, The University of Tokyo, 1-1-1 Yayoi, Bunkyo, Tokyo 113-8657, Japan
[2]Institute of Fruit Tree and Tea Science, National Agriculture and Food Research Organization (NARO), Okitsu Nakacho, Shimizu, Shizuoka 424-0292, Japan
[3]Genome Informatics Laboratory, National Institute of Genetics, Research Organization of Information and Systems, Yata 1111, Mishima, Shizuoka 411-8540, Japan
[4]Institute of Fruit Tree and Tea Science, NARO, Fujimoto, Tsukuba, Ibaraki 305-8605, Japan
[5]Institute of Crop Science, NARO, 2-1-2 Kannondai, Tsukuba, Ibaraki 305-8518, Japan
[6]Institute of Crop Science, NARO, Ohwashi 1-2, Tsukuba, Ibaraki 305-8634, Japan
[7]Comparative Genomics Laboratory, National Institute of Genetics, Research Organization of Information and Systems, Yata 1111, Mishima, Shizuoka 411-8540, Japan
[8]Advanced Genomics Center, National Institute of Genetics, Research Organization of Information and Systems, Yata 1111, Mishima, Shizuoka 411-8540, Japan

*To whom correspondence should be addressed: E-mail: aiwata@mail.ecc.u-tokyo.ac.jp

Supplementary Tables S1-S6
Supplementary Figures S1-S11

Supplementary Table S1. Parental citrus population used in this study.

Vatiety No.	Variety	Strain	Type	Fruit weight group (Fig. 2)[a]
1	Akemi	(stock strain)	Hybrid cultivar	Others
2	Aki tangor	(stock strain)	Hybrid cultivar	Others
3	Allspice	(stock strain)	Hybrid cultivar	Others
4	Amaka	(stock strain)	Hybrid cultivar	Others
5	Amakusa	(stock strain)	Hybrid cultivar	Others
6	Ariake	(stock strain)	Hybrid cultivar	Others
7	Asumi	(stock strain)	Hybrid cultivar	Others
8	Benibae	(stock strain)	Hybrid cultivar	Others
9	Ehime Kashi No.28	(stock strain)	Hybrid cultivar	Others
10	Encore	(stock strain)	Hybrid cultivar	Small group
11	Fairchild	(stock strain)	Hybrid cultivar	Small group
12	Fortune	(stock strain)	Hybrid cultivar	Small group
13	Harehime	(stock strain)	Hybrid cultivar	Others
14	Hareyaka	(stock strain)	Hybrid cultivar	Small group
15	Haruhi	(stock strain)	Hybrid cultivar	Others
16	Harumi	(stock strain)	Hybrid cultivar	Others
17	Hayaka	(stock strain)	Hybrid cultivar	Others
18	Kankitsu Chukanbohon Nou 5 Gou	(stock strain)	Hybrid cultivar	Small group
19	Kankitsu Chukanbohon Nou 6 Gou	(stock strain)	Hybrid cultivar	Others
20	Kanpei	(stock strain)	Hybrid cultivar	Others
21	Kara	(stock strain)	Hybrid cultivar	Others
22	Kiyomi	(stock strain)	Hybrid cultivar	Others
23	Lee	(stock strain)	Hybrid cultivar	Small group
24	Mihaya	(stock strain)	Hybrid cultivar	Others
25	Mihocore	(stock strain)	Hybrid cultivar	Others
26	Nanko	(stock strain)	Hybrid cultivar	Others
27	Nishinokaori	(stock strain)	Hybrid cultivar	Others
28	Nova	(stock strain)	Hybrid cultivar	Small group
29	Osceola	(stock strain)	Hybrid cultivar	Small group
30	Page	(stock strain)	Hybrid cultivar	Small group
31	Reiko	(stock strain)	Hybrid cultivar	Others
32	Robinson	(stock strain)	Hybrid cultivar	Small group
33	Saga mandarin	(stock strain)	Hybrid cultivar	Others
34	Seiho	(stock strain)	Hybrid cultivar	Others
35	Seinannohikari	(stock strain)	Hybrid cultivar	Others
36	Seminole	(stock strain)	Hybrid cultivar	Others
37	Setoka	(stock strain)	Hybrid cultivar	Others
38	Setomi	(stock strain)	Hybrid cultivar	Others
39	Shiranuhi	(stock strain)	Hybrid cultivar	Others
40	Southern Red	(stock strain)	Hybrid cultivar	Others
41	Southern Yellow	(stock strain)	Hybrid cultivar	Large group
42	Sweet Spring	(stock strain)	Hybrid cultivar	Others
43	Tamami	(stock strain)	Hybrid cultivar	Others
44	Tsunokagayaki	(stock strain)	Hybrid cultivar	Others
45	Tsunokaori	(stock strain)	Hybrid cultivar	Others
46	Tsunonozomi	(stock strain)	Hybrid cultivar	Others
47	Wilking	(stock strain)	Hybrid cultivar	Small group
48	Youkou	(stock strain)	Hybrid cultivar	Others
49	E-647	(stock strain)	Breeding/selected strains	Others
50	EnOw21	(stock strain)	Breeding/selected strains	Others

Supplementary Table S1. (*Continued*)

Vatiety No.	Variety	Strain	Type	Fruit weight group (Fig. 2)[a]
51	HF9	(stock strain)	Breeding/selected strains	Others
52	JHG	(stock strain)	Breeding/selected strains	Large group
53	Kuchinotsu 18	(stock strain)	Breeding/selected strains	Others
54	Kuchinotsu 27	(stock strain)	Breeding/selected strains	Others
55	Kuchinotsu 28	(stock strain)	Breeding/selected strains	Others
56	Kuchinotsu 33	(stock strain)	Breeding/selected strains	Others
57	Kuchinotsu 35	(stock strain)	Breeding/selected strains	Others
58	Kuchinotsu 36	(stock strain)	Breeding/selected strains	Others
59	Kuchinotsu 38	(stock strain)	Breeding/selected strains	Others
60	Kuchinotsu 39	(stock strain)	Breeding/selected strains	Others
61	Kuchinotsu 40	(stock strain)	Breeding/selected strains	Others
62	Kuchinotsu 49	(stock strain)	Breeding/selected strains	Others
63	Kuchinotsu 51	(stock strain)	Breeding/selected strains	Others
64	Kuchinotsu 52	(stock strain)	Breeding/selected strains	Others
65	KyEn4	(stock strain)	Breeding/selected strains	Others
66	KyEn5	(stock strain)	Breeding/selected strains	Others
67	KyOw14	(stock strain)	Breeding/selected strains	Others
68	KyOw21	(stock strain)	Breeding/selected strains	Others
69	KyOw21xAriake22	(stock strain)	Breeding/selected strains	Others
70	KyOw21xD4	(stock strain)	Breeding/selected strains	Others
71	LeeAo9	(stock strain)	Breeding/selected strains	Others
72	M5	(stock strain)	Breeding/selected strains	Others
73	Murcott	(stock strain)	Breeding/selected strains	Others
74	No.1011	(stock strain)	Breeding/selected strains	Others
75	No.1408	(stock strain)	Breeding/selected strains	Others
76	Okitsu 46	(stock strain)	Breeding/selected strains	Others
77	Okitsu 56	(stock strain)	Breeding/selected strains	Small group
78	Okitsu 57	(stock strain)	Breeding/selected strains	Others
79	Okitsu 59	(stock strain)	Breeding/selected strains	Others
80	Okitsu 60	(stock strain)	Breeding/selected strains	Others
81	Okitsu 62	(stock strain)	Breeding/selected strains	Others
82	Okitsu 63	(stock strain)	Breeding/selected strains	Others
83	Okitsu 67	(stock strain)	Breeding/selected strains	Others
84	SBxHg	(stock strain)	Breeding/selected strains	Large group
85	u-22	(stock strain)	Breeding/selected strains	Others
86	980389	(stock strain)	Breeding/selected strains	Others
87	010331	(stock strain)	Breeding/selected strains	Others
88	031045	(stock strain)	Breeding/selected strains	Others
89	2700xOIyo25	(stock strain)	Breeding/selected strains	Others
90	Banpeiyu	(stock strain)	Indigenous variety	Large group
91	Clementine	(stock strain)	Indigenous variety	Small group
92	Dancy	(stock strain)	Indigenous variety	Small group
93	Egami buntan	(stock strain)	Indigenous variety	Large group
94	Grapefruit	Marsh[b]	Indigenous variety	Others
95	Haruka	(stock strain)	Indigenous variety	Large group
96	Hassaku	(stock strain)	Indigenous variety	Large group
97	Hirado buntan	(stock strain)	Indigenous variety	Large group
98	Hyuganatsu	(stock strain)	Indigenous variety	Large group
99	Iyo	Miyauchi	Indigenous variety	Others
100	Kawachi bankan	(stock strain)	Indigenous variety	Large group
101	King	(stock strain)	Indigenous variety	Others
102	Kishu	Mukaku Kishu (seedless Kishu)	Indigenous variety	Small group
103	Mato buntan	(stock strain)	Indigenous variety	Large group
104	Mediterranean mandarin	(stock strain)	Indigenous variety	Small group
105	Natsudaidai	Kawano	Indigenous variety	Large group
106	Ponkan	Yoshida[b]	Indigenous variety	Small group
107	Satsuma	Miyagawa[b]	Indigenous variety	Others
108	Soren tangelo	(stock strain)	Indigenous variety	Large group
109	Sweet orange	Trovita[b]	Indigenous variety	Others
110	Tankan	Tarumizu1gou[b]	Indigenous variety	Small group
111	Yuge hyoukan	(stock strain)	Indigenous variety	Large group

[a] Large or Small fruit groups (Fig. 2) contained mainly pummelos or mandarins, respectively, while the remainders were defined as Others.

[b] Marsh, Yoshida, Miyagawa, Trovita, and Tarumizu1gou were used as representative mutants of Satsuma, ponkan, sweet orange, tankan, and grapefruit, respectively.

Supplementary Table S2. Breeding citrus population used in this study.

Family No.	Female	Vatiety No.	Male	Vatiety No.	Number of genotypes
1	Tamami	43	Shiranuhi	39	26
2	No.1011	74	Tsunonozomi	46	25
3	Tamami	43	Mihaya	24	26
4	980389	86	Shiranuhi	39	24
5	Mihaya	24	Kanpei	20	10
6	JHG	52	Southern Yellow	41	13
7	Kuchinotsu 36	58	Kankitsu Chukanbohon Nou 6 Gou	19	18
8	Ehime Kashi No.28	9	Okitsu 56	77	25
9	Satsuma Miyagawa	107	Benibae	8	25
10	Tankan T-132*	110	Setomi	38	18
11	KyEn5	66	Setomi	38	25
12	Harehime	13	Setoka	37	21
13	Kiyomi	22	Okitsu 57	78	21
14	Kuchinotsu 49	62	Seinannohikari	35	25
15	Ehime Kashi No.28	9	Hyuganatsu	98	25
16	EnOw21	50	Nanko	26	18
17	Harehime	13	King	101	16
18	Okitsu 46	76	Seinannohikari	35	13
19	Sweet Spring	42	Soren tangelo	108	11
20	Hyuganatsu	98	Southern Yellow	41	19
21	Tsunokagayaki	44	Kara	21	24
22	KyOw14	67	Okitsu 57	78	23
23	Harehime	13	Kanpei	20	26
24	KyOw14	67	San Jacinto*	-	7
25	Ehime Kashi No.28	9	Seinannohikari	35	25
26	Sweet Spring	42	Grapefruit triumph	94	17
27	Kuchinotsu 36	58	Kara	21	25
28	Okitsu 59	79	Okitsu 56	77	15
29	Asumi	7	Okitsu 56	77	25
30	Kuchinotsu 18	53	Harumi	16	22
31	Kawachi bankan	100	Shiranuhi	39	6
32	Hyuganatsu	98	Swet orange Trovita	109	20
33	Kiyomi	22	Grapefruit Redblush*	94	9
34	KyOw14	67	Kuchinotsu 51	63	23
35	Tsunonozomi	46	Satsuma Hinosayaka*	107	5

*Varieties not included in the parental citrus population (Supplementary Table S1).

Supplementary Table S3. Proportions of variance explained by the top three peak SNPs with high $-\log_{10}(p)$ values.

Proportions of variance explained of the top three peak SNPs detected by GWAS (red points in Supplementary Fig. S5) were estimated by the multiple linear regression (MLR) model.

	Weight	Appear	Shape	FruH	ColorP	SmoothP	Peeling	Aroma	ColorF	FleH	Juicy	FirmLM	Seed	Bitter	Taste	Brix	Acid
Proportion of variance explained	0.44	0.13	0.22	0.38	0.55	0.28	0.35	0.07	0.73	0.17	0.06	0.54	0.38	0.39	0.12	0.03	0.13

Supplementary Table S4. Significant SNPs detected by GWAS in the combined population.

Trait	SNP ID	Chromosome	Position (bp)	$-\log_{10}(p)$ value	Minor allele frequency (MAF)
Weight	citrus0389_2_52	2	31764775	4.71	0.33
Weight	citrus10077_SNP3_14506	3	26309463	8.79	0.25
Weight	citrus0611_3_48	3	32951108	5.28	0.35
Weight	citrus11216_SNP3_16561	3	33117562	4.41	0.35
Weight	citrus11324_SNP3_16742	3	33714865	7.22	0.39
Weight	citrus12526_SNP3_18550	3	39343248	4.49	0.30
Weight	citrus15705_SNP3_22822	3	43702161	4.17	0.06
Weight	citrus17812_SNP3_25612	3	46314924	5.47	0.04
Weight	citrus4831_SNP4_7515	4	13082786	5.49	0.04
Weight	citrus5360_SNP4_8375	4	14684617	3.69	0.06
Weight	citrus6779_SNP4_10366	4	17889120	4.18	0.03
Weight	citrus1651_8_3	8	1334839	5.02	0.39
Weight	citrus1806_8_54	8	14892334	11.12	0.46
Weight	citrus4415_SNP9_6638	9	10412348	3.78	0.24
Weight	citrus9435_SNP9_15027	9	29560382	3.45	0.03
FruH	citrus3050_SNP3_3975	3	3730998	4.72	0.43
FruH	citrus3256_SNP3_4220	3	3984445	7.22	0.40
FruH	citrus0496_3_10	3	4194184	7.46	0.43
FruH	citrus0497_3_10	3	4218314	6.14	0.47
FruH	citrus0498_3_10	3	4232262	4.25	0.41
ColorP	citrus7098_SNP2_10647	2	23988550	5.19	0.18
ColorP	citrus0324_2_30	2	24450181	3.55	0.14
ColorP	citrus4831_SNP4_7515	4	13082786	3.43	0.04
ColorP	citrus5360_SNP4_8375	4	14684617	8.29	0.06
ColorP	citrus5476_SNP4_8549	4	14983635	3.42	0.10
ColorP	citrus6089_SNP4_9453	4	16277936	3.47	0.18
ColorP	citrus6272_SNP4_9719	4	16730388	3.36	0.18
ColorP	citrus6589_SNP4_10104	4	17489519	5.81	0.03
ColorP	citrus6746_SNP4_10322	4	17823543	5.65	0.22
ColorP	citrus0863_4_53	4	18167929	5.17	0.22
ColorP	citrus7008_SNP4_10668	4	18248844	6.79	0.22
ColorP	citrus7312_SNP4_10993	4	18492701	5.31	0.22
ColorP	citrus7578_SNP4_11322	4	18968137	3.38	0.22
ColorP	citrus7645_SNP4_11420	4	19103301	5.91	0.07
ColorP	citrus4605_SNP6_7490	6	18091744	4.15	0.06
ColorP	citrus4861_SNP6_7836	6	18439865	3.86	0.06
ColorP	citrus5705_SNP8_8801	8	18606324	4.72	0.21
Peeling	citrus3256_SNP3_4220	3	3984445	4.92	0.40
Peeling	citrus0496_3_10	3	4194184	4.61	0.43
Peeling	citrus0497_3_10	3	4218314	4.22	0.47
ColorF	citrus6695_SNP2_10044	2	23101093	3.72	0.29
ColorF	citrus6820_SNP2_10209	2	23338290	4.67	0.15
ColorF	citrus6855_SNP2_10322	2	23651414	5.97	0.20
ColorF	citrus7098_SNP2_10647	2	23988550	10.37	0.18
ColorF	citrus0324_2_30	2	24450181	5.85	0.14
ColorF	citrus7430_SNP2_11211	2	25231326	3.47	0.49
ColorF	citrus6589_SNP4_10104	4	17489519	8.07	0.03
ColorF	citrus7008_SNP4_10668	4	18248844	4.10	0.22
ColorF	citrus7645_SNP4_11420	4	19103301	4.63	0.07
ColorF	citrus0947_SNP6_1825	6	7359200	6.63	0.17
ColorF	citrus1701_SNP6_3102	6	10485823	4.77	0.16
ColorF	citrus4605_SNP6_7490	6	18091744	9.08	0.06
ColorF	citrus4861_SNP6_7836	6	18439865	6.91	0.06
FleH	citrus6350_SNP3_8386	3	8487047	4.57	0.18
FleH	citrus5592_SNP4_8686	4	15180049	4.82	0.32
Juicy	citrus5592_SNP4_8686	4	15180049	4.82	0.32
FirmLM	citrus17812_SNP3_25612	3	46314924	5.05	0.04
FirmLM	citrus18213_SNP3_26123	3	46704452	4.30	0.03
FirmLM	citrus18467_SNP3_26445	3	47011404	4.56	0.03
FirmLM	citrus3655_SNP4_5275	4	7939349	9.71	0.05
FirmLM	citrus7645_SNP4_11420	4	19103301	5.94	0.07
Seed	citrus3655_SNP4_5275	4	7939349	4.67	0.05
Bitter	citrus10194_SNP3_14669	3	26667163	3.77	0.24
Bitter	citrus5360_SNP4_8375	4	14684617	3.98	0.06
Bitter	citrus6779_SNP4_10366	4	17889120	4.31	0.03
Bitter	citrus0947_SNP6_1825	6	7359200	3.78	0.17
Bitter	citrus4605_SNP6_7490	6	18091744	3.78	0.06
Bitter	citrus4861_SNP6_7836	6	18439865	5.26	0.06
Acid	citrus5360_SNP4_8375	4	14684617	5.37	0.06

Supplementary Table S5. Phenotypic correlation in the parental and breeding citrus populations combined.

Phenotypic correlation was measured as a Pearson's correlation coefficient (r) between the phenotypic values of the traits.

	Weight	Appear	Shape	FruH	ColorP	SmoothP	Peeling	Aroma	ColorF	FleH	Juicy	FirmLM	Seed	Bitter	Taste	Brix	Acid
Weight	1.00	-0.31	0.02	0.20	-0.26	-0.02	0.23	-0.04	-0.29	0.18	0.09	0.22	0.03	0.18	-0.12	-0.23	0.01
Appear		1.00	-0.04	0.06	0.03	0.35	0.04	0.01	0.01	0.09	0.11	0.11	-0.02	-0.01	0.14	0.13	-0.03
Shape			1.00	-0.07	0.07	-0.03	-0.14	-0.02	0.09	0.03	0.02	-0.07	0.01	-0.05	0.01	-0.01	-0.01
FruH				1.00	-0.38	0.09	0.76	-0.07	-0.36	0.47	0.29	0.42	0.12	0.32	0.22	-0.17	0.27
ColorP					1.00	0.03	-0.34	-0.03	0.81	-0.16	-0.05	-0.33	-0.19	-0.31	-0.11	0.30	-0.35
SmoothP						1.00	0.06	0.04	0.01	0.15	0.18	0.14	-0.05	0.03	0.07	0.07	-0.22
Peeling							1.00	-0.02	-0.38	0.32	0.17	0.29	0.03	0.31	0.10	-0.09	0.25
Aroma								1.00	-0.03	0.16	0.18	-0.11	-0.11	0.00	0.00	0.04	-0.16
ColorF									1.00	-0.25	-0.16	-0.33	-0.21	-0.34	-0.18	0.34	-0.35
FleH										1.00	0.68	0.15	-0.07	0.15	0.31	-0.11	0.03
Juicy											1.00	0.11	-0.04	0.07	0.33	-0.08	-0.15
FirmLM												1.00	0.35	0.21	0.28	-0.26	0.12
Seed													1.00	0.15	0.10	-0.04	0.13
Bitter														1.00	0.20	-0.12	0.21
Taste															1.00	-0.29	0.17
Brix																1.00	-0.11
Acid																	1.00

Supplementary Table S6. Estimates of the additive (σ_a^2) and dominance (σ_d^2) genetic and residual (σ_E^2) variances.

Source	Weight	Appear	Shape	FruH	ColorP	SmoothP	Peeling	Aroma	ColorF	FleH	Juicy	FirmLM	Seed	Bitter	Taste	Brix	Acid
σ_a^2	10432.08	0.01	1.60	0.61	0.61	0.34	0.46	0.02	0.49	0.19	0.01	0.29	0.67	0.00	0.03	0.45	0.22
σ_d^2	6512.64	0.01	0.72	0.18	0.21	0.08	0.14	0.01	0.16	0.09	0.01	0.10	0.18	0.00	0.01	0.23	0.07
σ_e^2	7860.81	0.02	1.76	0.26	0.25	0.15	0.19	0.01	0.19	0.17	0.01	0.15	0.33	0.00	0.02	0.32	0.08
h^2	0.42	0.28	0.39	0.58	0.57	0.60	0.58	0.54	0.58	0.42	0.30	0.54	0.57	0.27	0.45	0.45	0.60

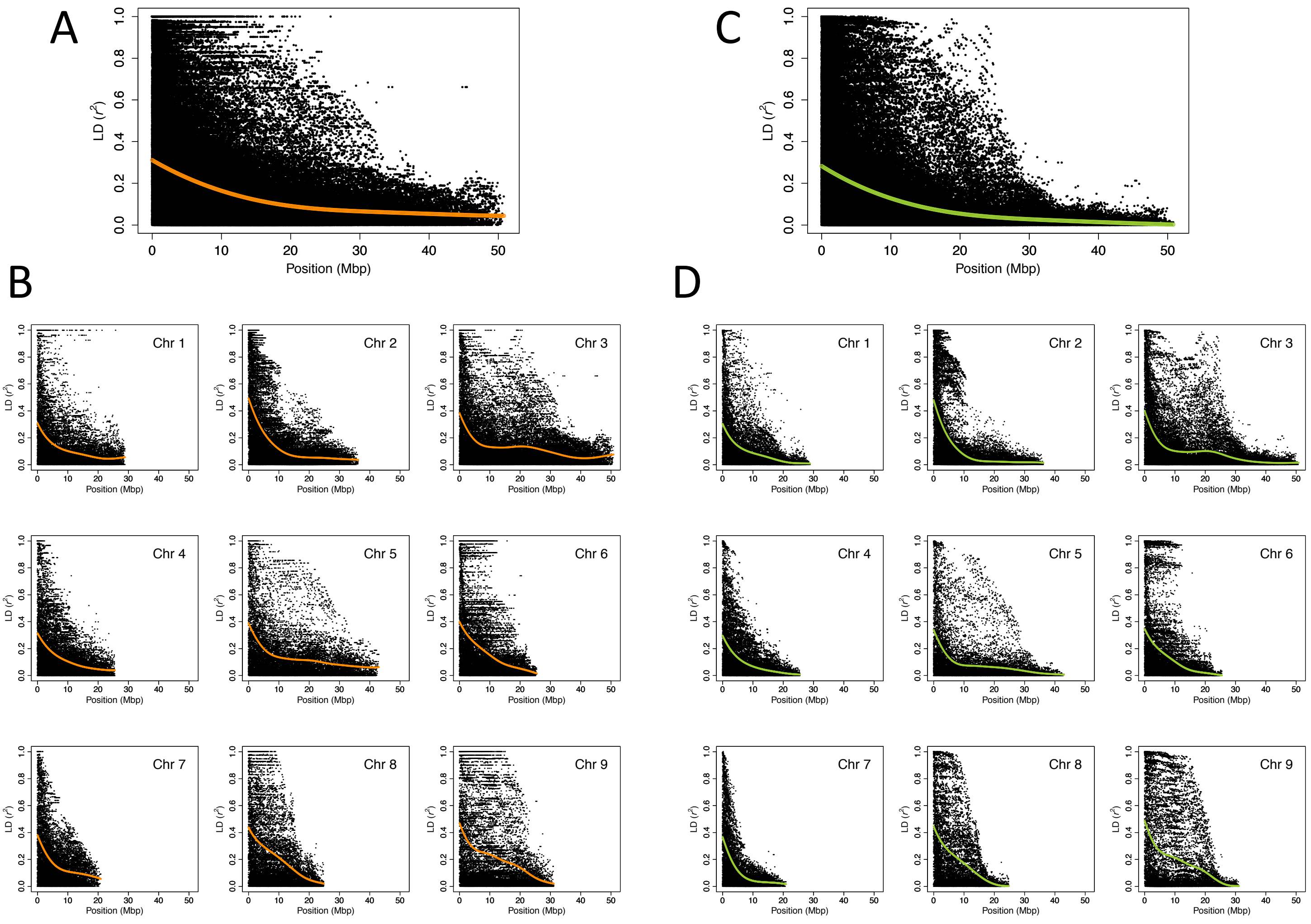

Supplementary Figure S1. LD values (r^2) between SNP pairs plotted against physical distances between the SNPs.
(A), (B) Parental population; (C), (D) Combined parental and breeding populations; (A), (C) The entire genome; (B), (D) Individual chromosomes. Colored curves show local polynomial smoothed plots with kernel weight.

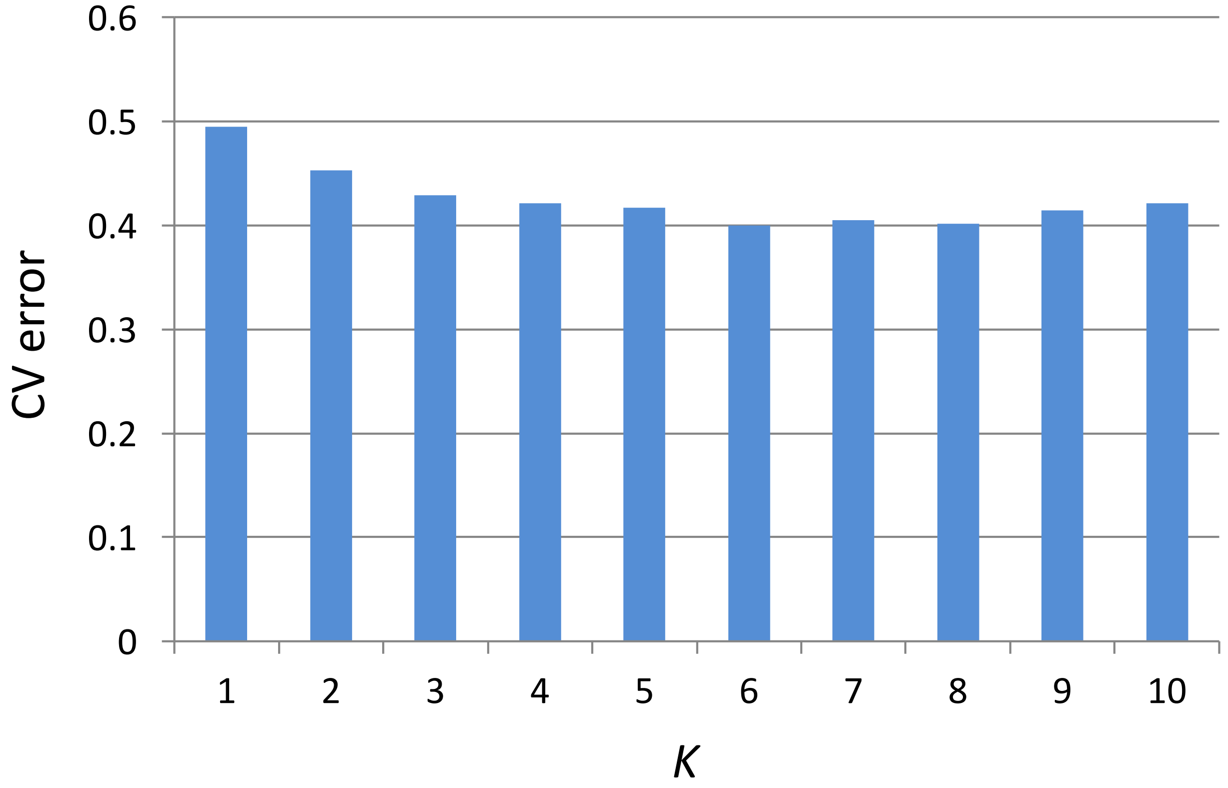

Supplementary Figure S2. Cross-validation (CV) errors returned by ADMIXTURE.

ADMIXTURE was used to examine the parental population with 1,841 SNPs.

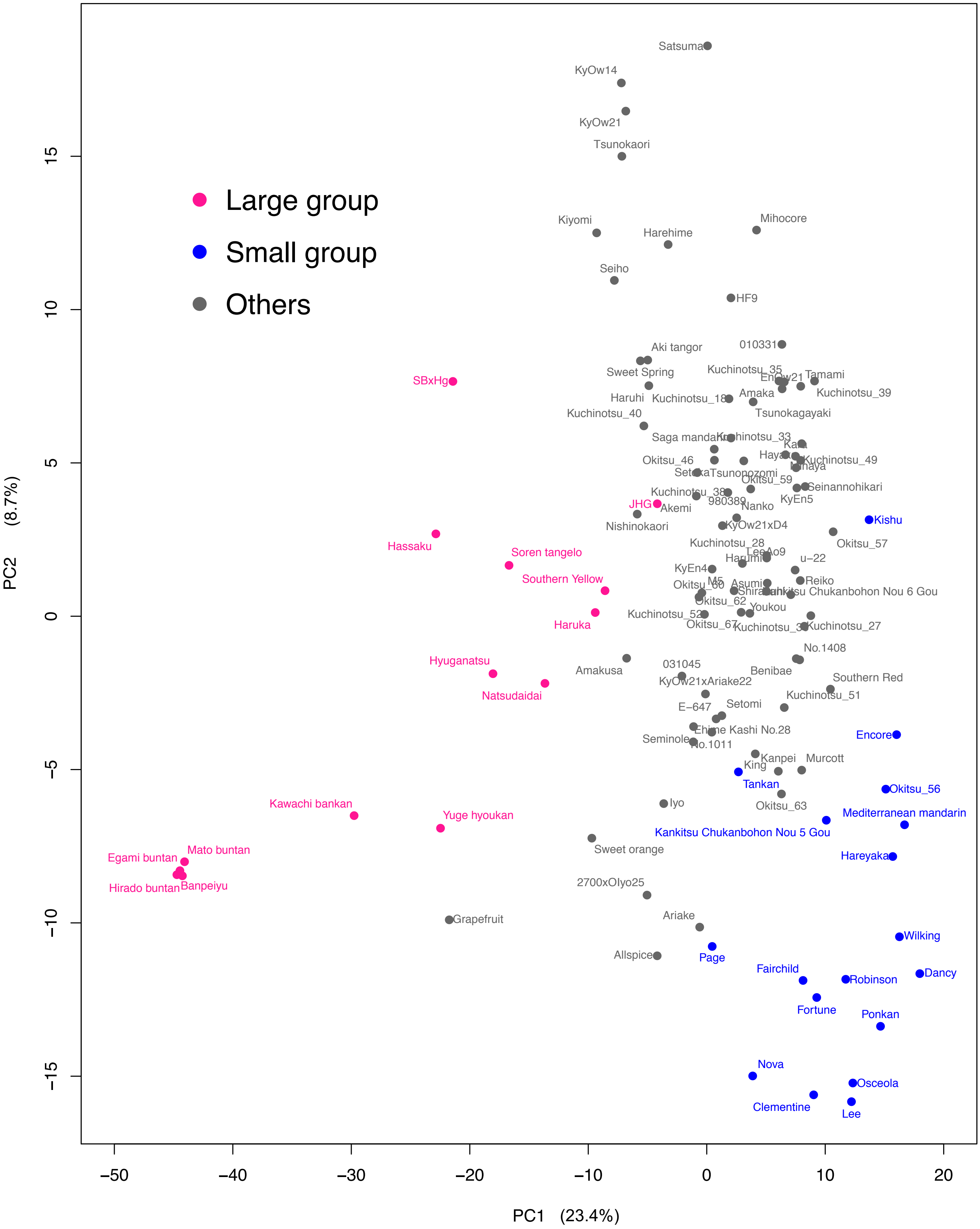

Supplementary Figure S3. Principal component analysis of the parental population.

Large group, accessions with large fruit weight; small group, accessions with small fruit weight (see Fig. 2C).

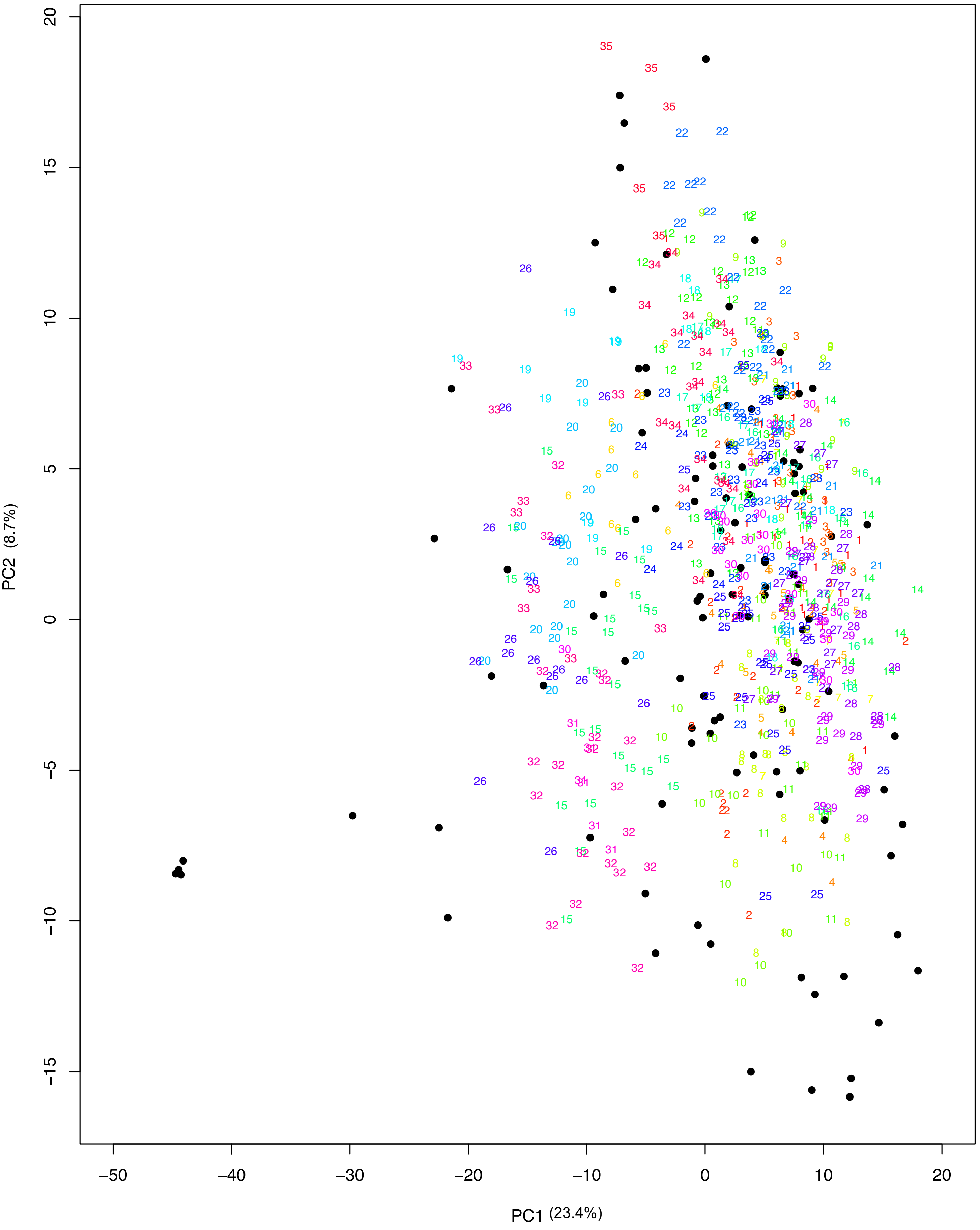

Supplementary Figure S4. Principal component analysis of parental and breeding populations.

Black circles indicate parental population. Numbers indicate the families of breeding population (see Supplementary Table S2) and colors correspond to the families.

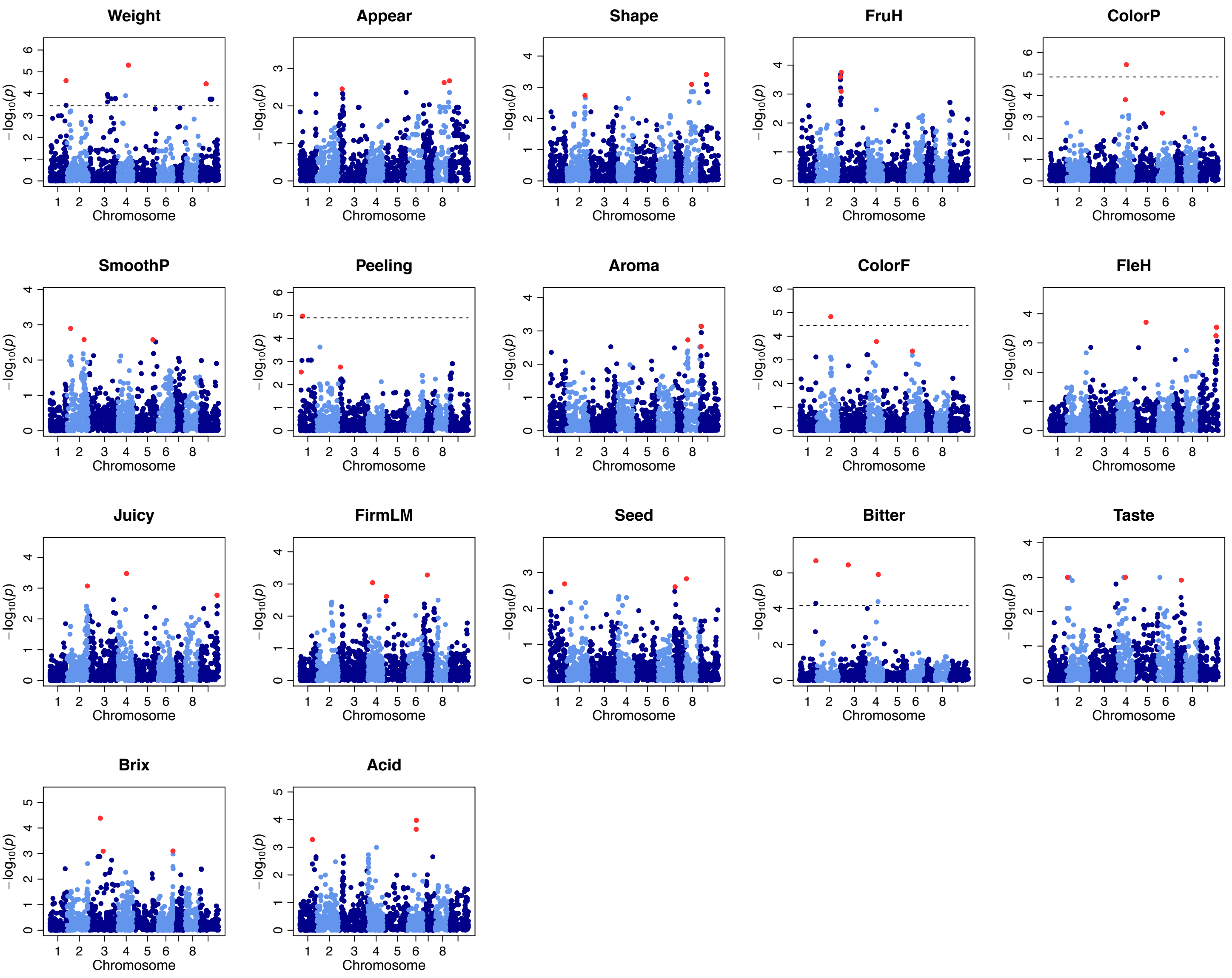

Supplementary Figure S5. Manhattan plots for 17 fruit quality traits in the parental population.

Mixed linear model used four principal components of the population structure as covariates (Supplementary Fig. S6). Dashed lines indicate a false discovery rate of 0.05. SNPs used for multiple linear regression (MLR) and GBLUP (RR) (Fig. 4) are shown in red. The proportions of variance explained of the SNPs were estimated by MLR model (Supplementary Table S3).

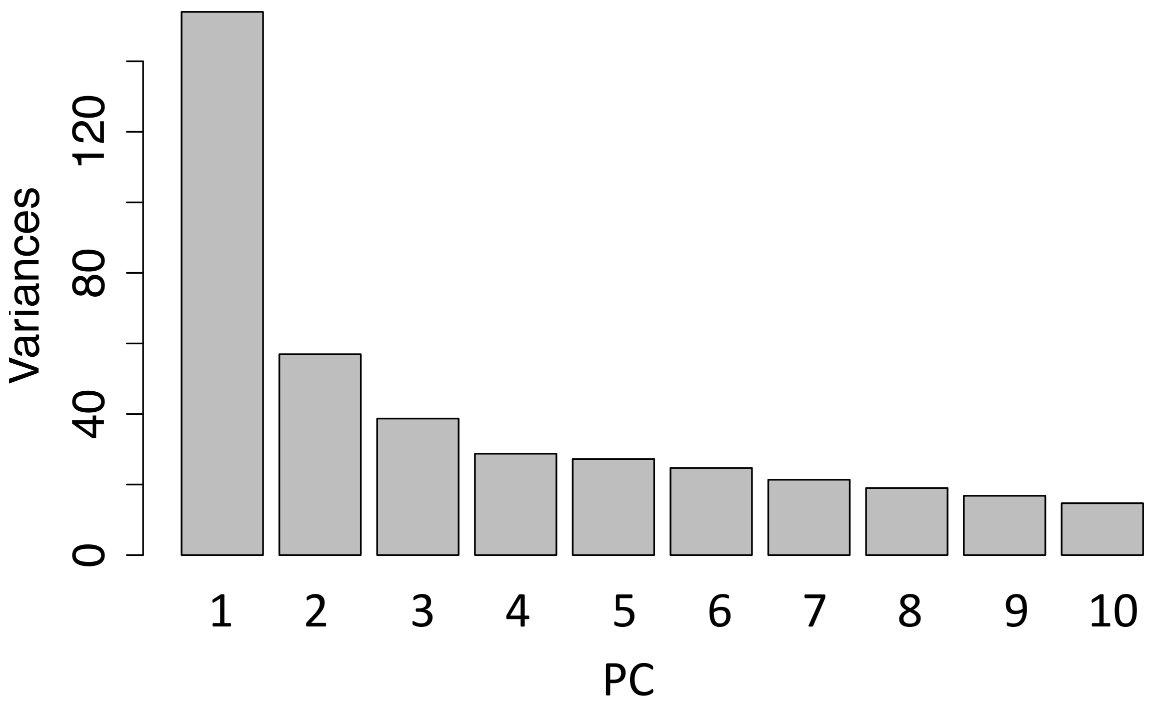

Supplementary Figure S6. Variances of principal component (PC) scores. Principal component analysis was used to examine the parental population with 1,841 SNPs.

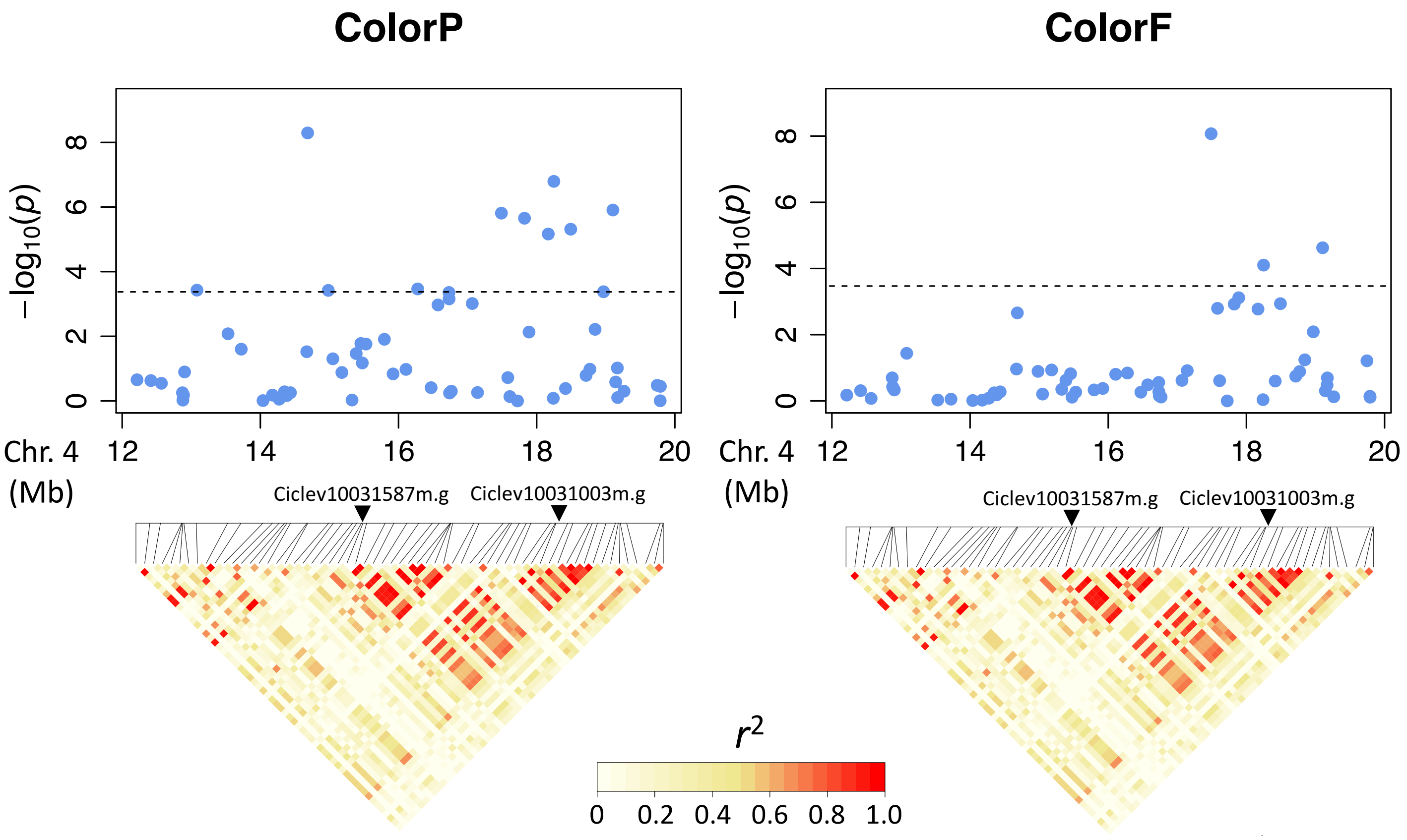

Supplementary Figure S7. Local Manhattan plots and LD heatmaps of the regions surrounding the peaks on chromosome 4 for the color of pericarp (ColorP) and color of flesh (ColorF) in the combined population. Dashed lines indicate a false discovery rate of 0.05. Arrowheads indicate the positions of the Ciclev10031587m.g and Ciclev10031003m.g genes.

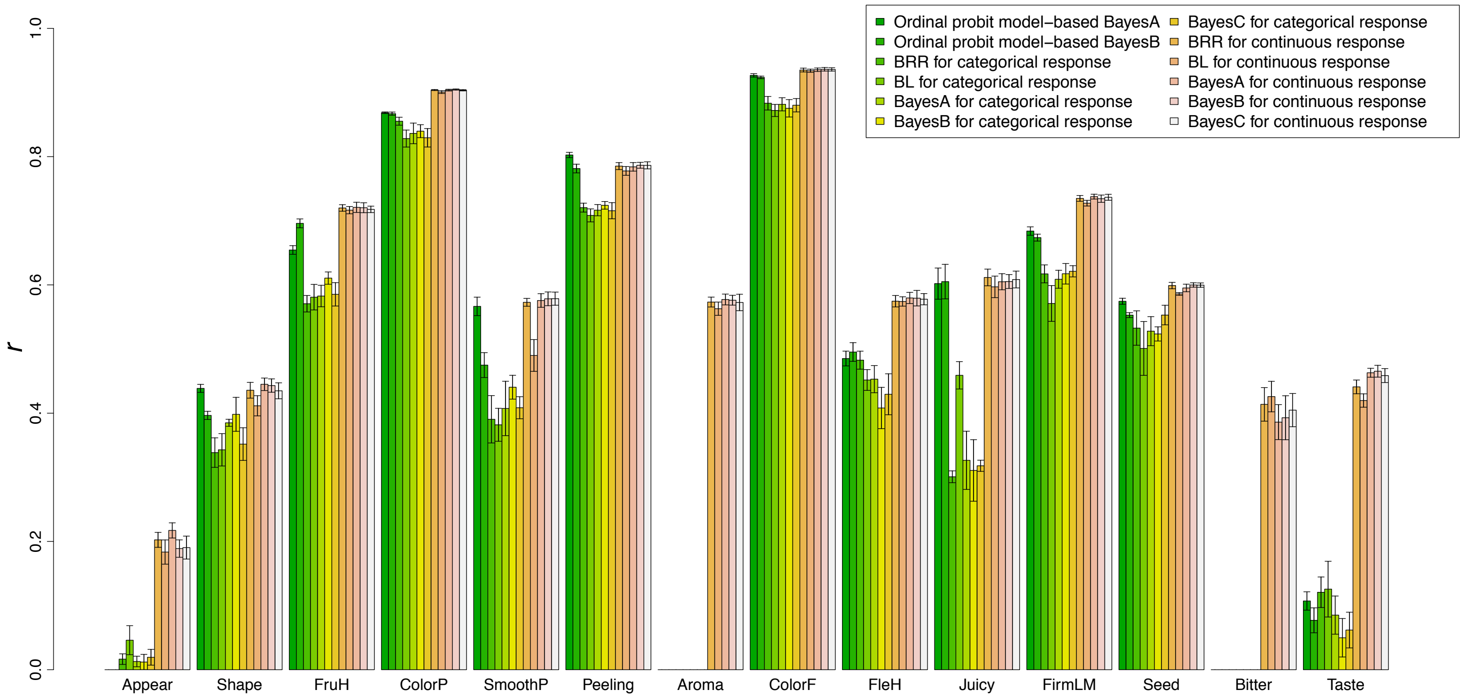

Supplementary Figure S8. Comparison of the prediction accuracy of the models for categorical responses and that for continuous responses. Accuracy was evaluated using 10-fold CV repeated 5 times and expressed as a Pearson's correlation coefficient (*r*) between predicted genotypic values and phenotypic values. BGLR for Aroma and Bitter could not be used because of their few ordinal values. BRR: Bayesian Ridge Regression, BL: Bayesian Lasso.

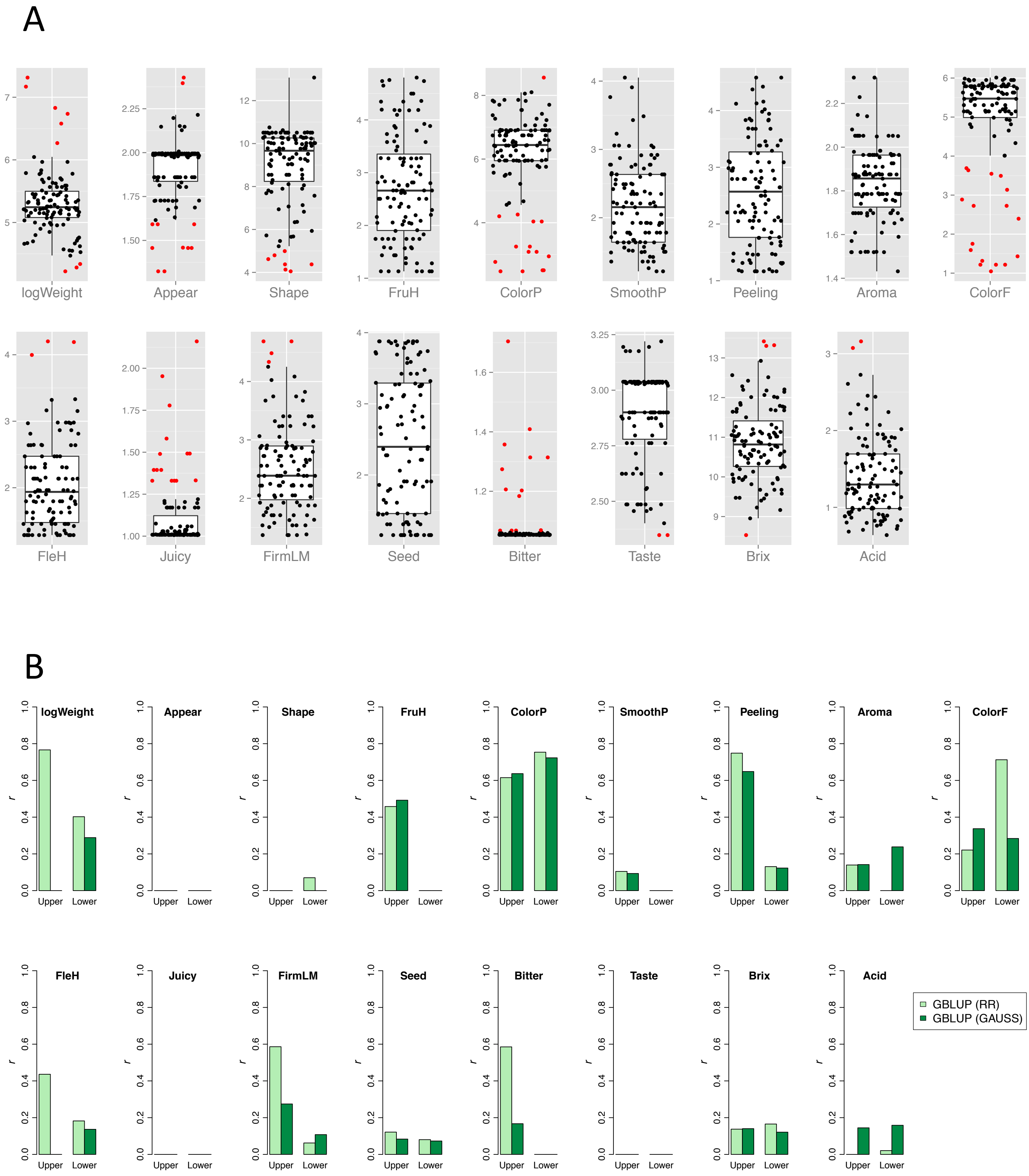

Supplementary Figure S9. Phenotypic variance and genomic prediction for the distribution tails of 17 fruit quality traits in the parental population.
(A) Jitter plots were superimposed onto the boxplots of phenotypic variance. Outliers estimated by boxplot analysis are shown in red. (B) Rank-ordered 4-fold CV of the parental population. Prediction accuracies of the upper and lower folds are shown. The accuracy was evaluated as a Pearson's correlation coefficient (*r*) between predicted genotypic values and phenotypic values. RR: linear ridge kernel regression, GAUSS: non-linear Gaussian kernel regression.

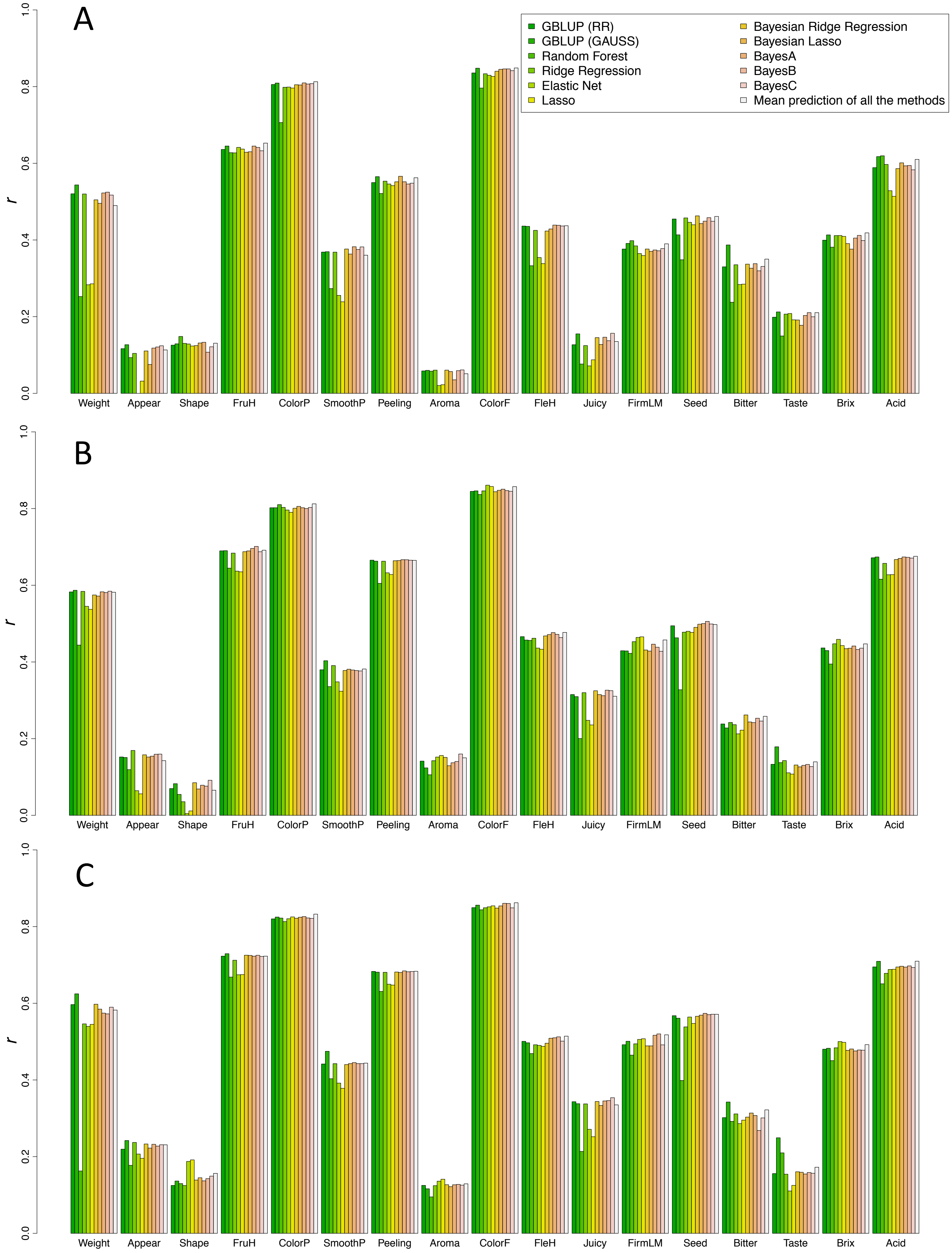

Supplementary Figure S10. Prediction accuracy of 12 methods in the breeding population.
Accuracy was measured as a Pearson's correlation coefficient (*r*) between predicted genotypic values and phenotypic values. (A) Parental population was used as a training population for the prediction models. (B) Breeding population (with one family excluded) was used as a training population and the phenotype of the excluded family was predicted. (C) Parental and breeding populations combined (with one family excluded) was used as a training population and the phenotype of the excluded family was predicted.

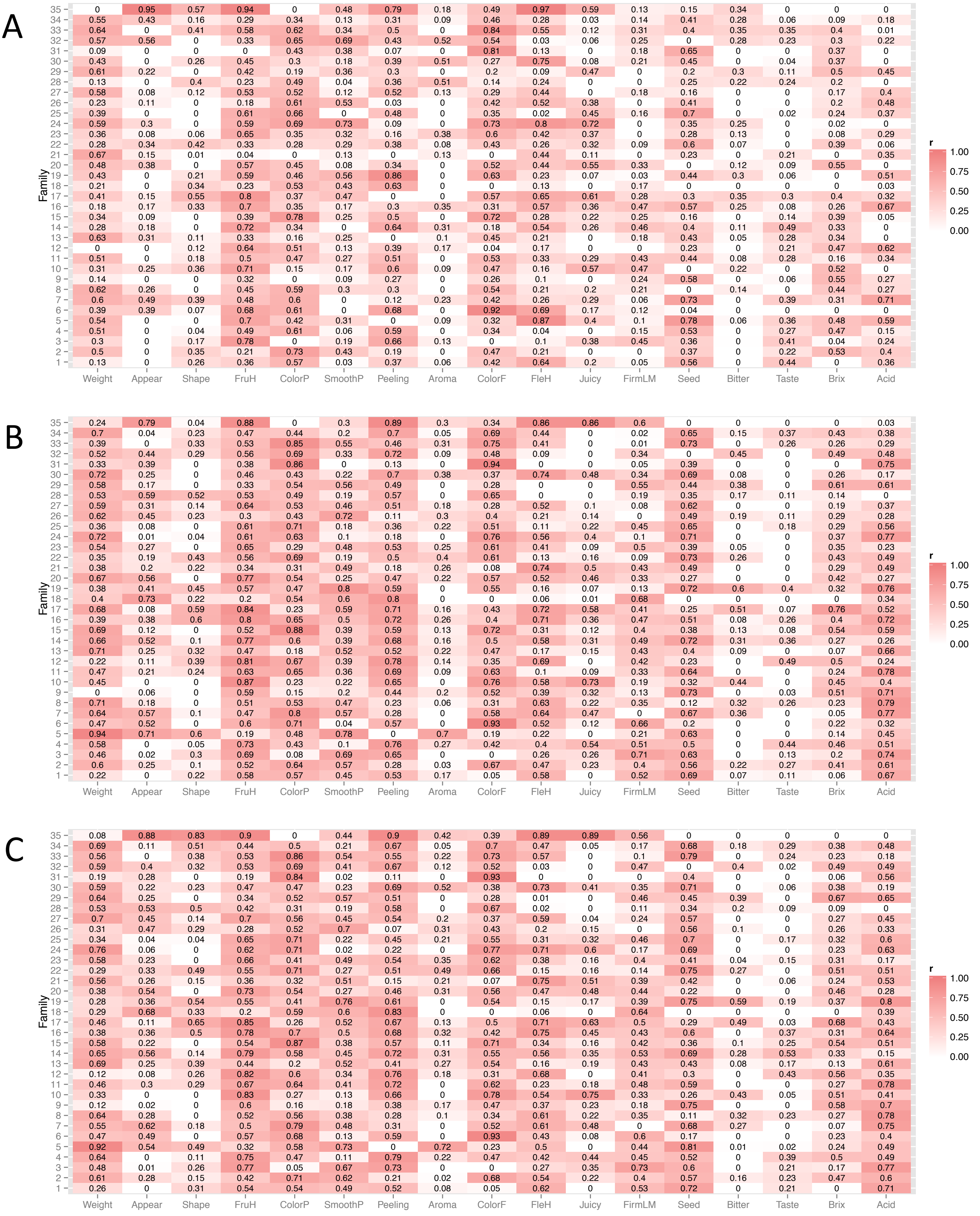

Supplementary Fig S11. Prediction accuracy in each family of the breeding population.
The mean prediction accuracy of all methods is shown as a Pearson's correlation coefficient (r) between predicted genotypic values and phenotypic values. (A) Parental population was used as a training population for the prediction models. (B) Breeding population (with one family excluded) was used as a training population and the phenotype of the excluded family was predicted. (C) Parental and breeding populations combined (with one family excluded) were used as a training population and the phenotype of the excluded family was predicted.
